# Helping koalas battle disease – Recent advances in *Chlamydia* and koala retrovirus (KoRV) disease understanding and treatment in koalas

**DOI:** 10.1093/femsre/fuaa024

**Published:** 2020-06-18

**Authors:** Bonnie L Quigley, Peter Timms

**Affiliations:** Genecology Research Centre, University of the Sunshine Coast, 90 Sippy Downs Drive, Sippy Downs, Queensland, 4556, Australia; Genecology Research Centre, University of the Sunshine Coast, 90 Sippy Downs Drive, Sippy Downs, Queensland, 4556, Australia

**Keywords:** *Chlamydia*, *Chlamydia pecorum*, koala retrovirus, KoRV, koala, *Phascolarctos cinereus*

## Abstract

The iconic Australian marsupial, the koala (*Phascolarctos cinereus*), has suffered dramatic population declines as a result of habitat loss and fragmentation, disease, vehicle collision mortality, dog attacks, bushfires and climate change. In 2012, koalas were officially declared vulnerable by the Australian government and listed as a threatened species. In response, research into diseases affecting koalas has expanded rapidly. The two major pathogens affecting koalas are *Chlamydia pecorum*, leading to chlamydial disease and koala retrovirus (KoRV). In the last eight years, these pathogens and their diseases have received focused study regarding their sources, genetics, prevalence, disease presentation and transmission. This has led to vast improvements in pathogen detection and treatment, including the ongoing development of vaccines for each as a management and control strategy. This review will summarize and highlight the important advances made in understanding and combating *C. pecorum* and KoRV in koalas, since they were declared a threatened species. With complementary advances having also been made from the koala genome sequence and in our understanding of the koala immune system, we are primed to make a significant positive impact on koala health into the future.

## Teaser

Recent advances in understanding the two major pathogens of koalas, *Chlamydia pecorum* and koala retrovirus (KoRV), have benefited both koala conservation and general chlamydial and retroviral research fields.

## INTRODUCTION

**Figure 1.**
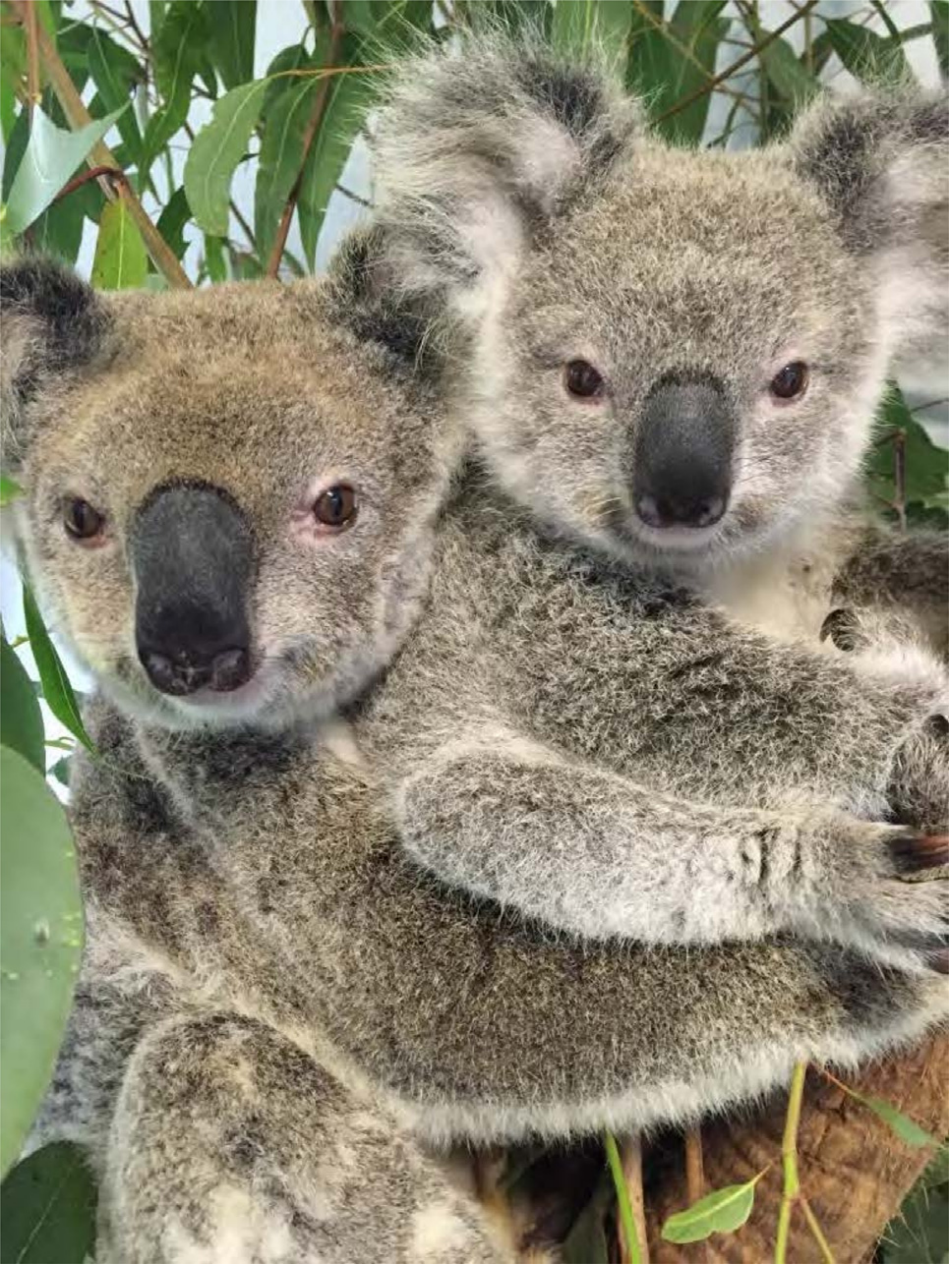
Koalas (*Phascolarctos cinereus*). Photo credit to Endeavour Veterinary Ecology.

**Figure 2.**
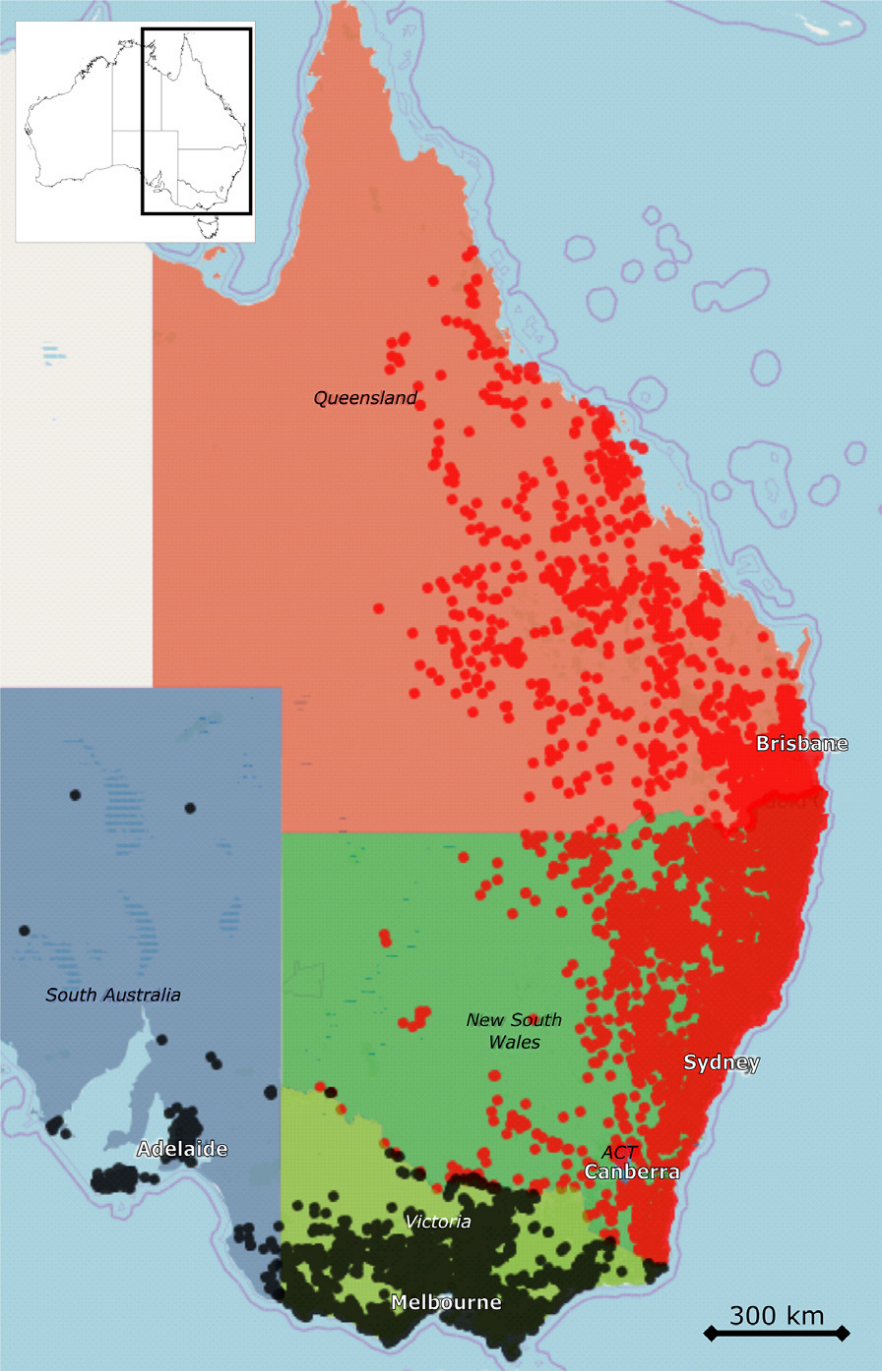
Current range of koalas in Australia. Locations where koalas are listed as vulnerable (from Queensland, New South Wales and Australian Capital Territory (ACT)) are indicated by red dots while locations where koalas do not have a conservation listing (Victoria and South Australia) are indicated by black dots. Map and koala location data are from Atlas of Living Australia website at http://www.ala.org.au. Accessed 10 February 2020.

Disease is a major conservation challenge for the well-loved Australian marsupial, the koala (*Phascolarctos cinereus*) (Fig. 1). Once believed to number in the millions (Phillips and Service 1990), experts now estimate the koala population in Australia to be approximately 330 000 animals, with major and continued population decline anticipated in the northern half of their range (Melzer *et al*. 2000; McAlpine *et al*. 2015; Adams-Hosking *et al*. 2016; Beyer *et al*. 2018). This has resulted in koalas from the northern half of Australia being added to the Australian Environmental Protection Biodiversity Conservation Act in 2012 (Australia 2011) (Fig. 2) and all koalas being listed as “vulnerable” on the Red List of Threatened Species worldwide (Woinarski and Burbidge 2016). While multiple threats have been identified as impacting koala populations, including habitat degradation/loss, disease, dog attacks, motor vehicle strikes, bushfires and climate change, the significant impact of disease, particularly from *Chlamydia* and koala retrovirus (KoRV), is routinely highlighted (Australia 2011; Hemming *et al*. 2018). In response, targeted research into koala diseases has expanded rapidly since 2012. Major advances in understanding the source, genetics, prevalence, disease presentation and transmission of the two major koala pathogens, *Chlamydia pecorum* and KoRV, has led to complementary advances in pathogen detection and treatment. The overall progress that has been made in koala disease research since 2012 has dramatically improved our knowledge of the koala disease landscape and equipped us with the tools to make a significant improvement in koala conservation moving forward.

## CHLAMYDIA PECORUM

*Chlamydia pecorum* is the bacterium that causes chlamydial disease in koalas. While *Chlamydia pneumoniae* has also been reported in koalas, recent molecular surveys have detected virtually no *C. pneumoniae* in koalas (Burach *et al*. 2014; Johnston *et al*. 2015; Patterson *et al*. 2015; Legione *et al*. 2016b; Santamaria and Schlagloth 2016; Hulse *et al*. 2018; Palmieri *et al*. 2019), indicating that this species is currently not a major contributor to disease. Like all *Chlamydia*, *C. pecorum* has a unique biphasic life cycle. When extracellular, the chlamydiae exist as metabolically inactive infectious particles known as elementary bodies (EBs). EBs are taken up by susceptible host cells into inclusions, where they prevent phagosome-lysozyme fusion and replicate in this intracellular compartment as reticulate bodies (RBs). Once enough RBs have formed, cells convert back into EBs to lyse the cell and infect other cells.

**Figure 3.**
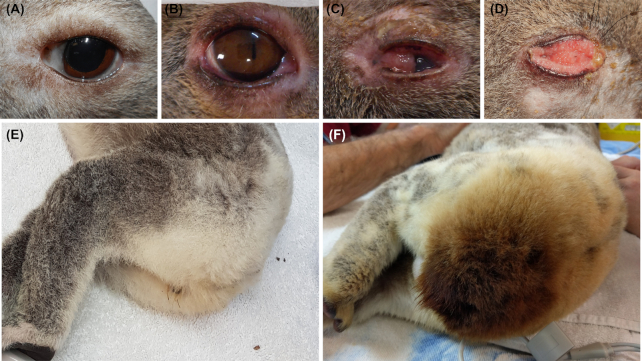
*Chlamydia pecorum* disease in koalas. **(A)**, Healthy koala eye, **(B)**, Mild conjunctivitis, **(C)**, Moderate conjunctivitis/keratoconjunctivitis with chemosis, purulent periocular discharge and a mildly proliferative prolapsed nictitating membrane, **(D)**, Severe conjunctivitis with purulent discharge, marked proliferation of conjunctival tissues, completing obscuring the eye, **(E)**, Healthy koala rump, **(F)**, Koala with cystitis, rump fur stained by urine due to incontinence (“wet bottom”). Photo credit to Australia Zoo Wildlife Hospital.

*C. pecorum* is classically associated with ocular and urogenital disease in koalas (Fig. 3). When the infection establishes in the conjunctiva of the eye, chronic conjunctivitis and keratoconjunctivitis can lead to corneal scarring and eventual blindness (Fig. 3A–D) (Blanshard and Bodley 2008; Jelocnik *et al*. 2019). When the infection establishes in the urinary tract (including the urethra, bladder, ureters and kidneys), the accompanying urethritis, cystitis, ureteritis and/or pyelonephritis can cause severe pain, polyuria and/or urinary incontinence leading to “wet-bottom” (urine staining the rump fur, Fig. 3E and F), as well as skin ulceration and secondary infection (Polkinghorne, Hanger and Timms 2013; Jelocnik *et al*. 2019). Finally, when infection establishes in the reproductive tract, inflammation in both females (eg. salpingitis, endometritis, vaginitis) and males (eg. epididymitis, orchitis, urethritis) can lead to scarring and infertility (Blanshard and Bodley 2008; Johnston *et al*. 2015; Palmieri *et al*. 2019). *C. pecorum* infection is, however, not only limited to the classic body sites within the koala, with recent reports implicating this pathogen in fatal pneumonia (Mackie *et al*. 2016), polyarthritis (Burnard, Gillett and Polkinghorne 2018) and colonization of the gastrointestinal tract (Burach *et al*. 2014; Wedrowicz *et al*. 2016; Phillips *et al*. 2018).

### Origin of *C. pecorum* in koalas

Although records of koalas before and during early European settlement in Australia are sparse, there is a general belief that chlamydiosis has been a component of koalas’ natural history (Phillips 2000). However, recent molecular epidemiology investigations of *C. pecorum* across several animal hosts have introduced the hypothesis that at least some koala *Chlamydia* infections may be spill-over events from introduced domestic livestock. Both multilocus sequence typing (MLST) and whole genome comparisons of *C. pecorum* have detected genetically similar strains between koalas and sheep (Jelocnik *et al*. 2013, 2014; Bachmann *et al*. 2015). Additionally, two koalas from French Island, Victoria (VIC) (a closed island koala population believed to be *Chlamydia*-free) have now been found to be infected with *C. pecorum* strains more closely related to known cattle (which are farmed on the island) and pig livestock strains compared to known koala strains from the mainland (Legione *et al*.2016a). More epidemiological tracking is needed to clarify whether these apparent livestock/koala spill-over events are common and if these events are having an appreciable impact on koala disease. However, these findings do highlight that *C. pecorum* has a broad host range and the introduction of domestic livestock to Australia cannot be ruled out as a source of chlamydial infection for native animals like the koala.

### Advances in genetic understanding of *C. pecorum*

**Figure 4.**
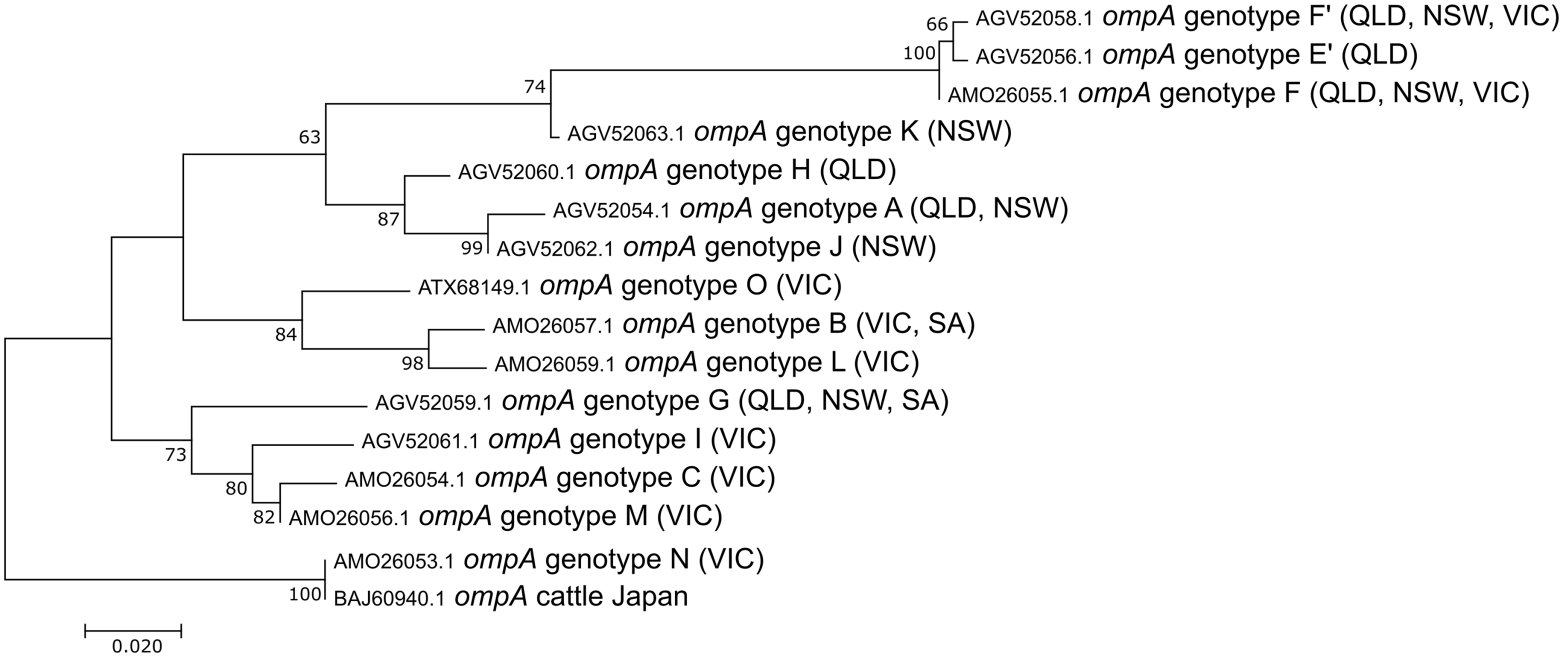
Maximum likelihood tree diagramming the relatedness of *Chlamydia pecorum ompA* genotypes. The Jones, Taylor, Thornton matrix model was used to construct the tree with 100 bootstrap confidence. Bootstrap values above 60 are shown on the nodes. A *C. pecorum* isolate from cattle in Japan was included as an outlier. Australian states with koalas positive for each genotype are indicated in the genotype label. QLD – Queensland, NSW – New South Wales, VIC – Victoria, SA – South Australia.

In the last decade, advances in whole genome sequencing and bioinformatics have allowed for new analyses of *C. pecorum* strains infecting koalas. From the individual gene target perspective, the Major Outer Membrane Protein (MOMP), coded by the *ompA* gene, has remained a favorite target for strain typing chlamydial diversity in koala populations (Kollipara *et al*. 2013c; Legione *et al*.2016b; Nyari *et al*. 2017; Wedrowicz *et al*. 2018). Surveys from across Australia have identified 15 unique *ompA* genotypes in koalas (Fig. 4). Based on the phylogeny of the *ompA* gene, there appears to be a general separation of *ompA* genotypes into two major groups; one group consisting of two clades detected mostly in the northern half of the country (genotypes A, E’, F, F’, H, J and K) and another group consisting of two clades detected mostly in the southern half of the country (genotypes B, C, G, I, L, M, N, O) (Fig. 4). However, the geographical separation of the genotypes is not absolute, with genotypes F, F’ and G being detected in koalas from both northern (Queensland (QLD) and New South Wales (NSW)) and southern (VIC and South Australia (SA)) geographic ranges.

Concern that the *ompA* gene did not reliably reflect the genetic diversity and relationship of *C. pecorum* strains alone led to the development of a *C. pecorum* MLST scheme (Jelocnik *et al*. 2013). Targeting seven *C. pecorum* house-keeping genes, *enoA, oppA_3, gidA, hemN, hflX, fumC* and *gatA*, the MLST scheme has been used as an alternative system for *C. pecorum* strain typing (Jelocnik *et al*. 2014; Fernandez *et al*. 2019). Although not as widely utilized as the traditional *ompA* genotyping system, MLST typing of koala *C. pecorum* strains has allowed for greater comparison of koala isolates to other hosts around the world, underpinning new investigations in strain tracking and transmission between hosts. Alterative *C. pecorum* gene targets with variable tandem repeat regions, such as *incA* and ORF663, have also been investigated to characterize koala *C. pecorum* strains (Higgins *et al*. 2012; Mohamad *et al*. 2014). For both genes, initial analysis has revealed that koala *C. pecorum* strains do possess variable numbers of tandem repeats and that there may be a link between a higher number of repeats and less virulent strains (Higgins *et al*. 2012; Mohamad *et al*. 2014). However, with the continued advancement of whole genome analysis over single gene studies, non-*ompA* typing and targeting studies have trailed off in recent years.

Whole genome analysis of koala *C. pecorum* strains has advanced our understanding of several important genetic and biological properties of this koala pathogen. Sequencing has revealed that the polymorphic membrane protein (*pmp*) gene cluster and the plasticity zone of the *C. pecorum* genome are hot spots for single nucleotide polymorphisms (SNPs) between strains (Bachmann *et al*. 2015; Jelocnik *et al*. 2015). A plasmid with potential virulence genes has been detected in the majority of *C. pecorum* strains, with an association between plasmid carriage and disease outcomes starting to emerge (Jelocnik *et al*. 2015, 2016; Phillips *et al*. 2018). In addition, shotgun sequencing directly from clinical samples has revealed the presence of multiple distinct *C. pecorum* strains, often at the urogenital site, within an individual koala (Bachmann *et al*. 2015). This finding reminds us that infections are often complex within an animal and most simple typing schemes likely underrepresent the actual *C. pecorum* complexity present.

Finally, whole genome sequencing of *C. pecorum* strains initially illuminated an important biological difference between this species and its related human pathogen, *Chlamydia trachomatis*. *C. trachomatis* is a tryptophan auxotroph, having only the tryptophan synthase (*trpBA*) gene for the final conversion of indole to tryptophan (Ziklo *et al*. 2016b). This results in *C. trachomatis* being very sensitive to interferon-gamma (IFN-ɣ) mediated depletion of host tryptophan (Islam *et al*. 2018). Alternatively, genome sequencing revealed that *C. pecorum* possesses a nearly complete tryptophan biosynthesis operon (Mojica *et al*. 2011; Bachmann *et al*. 2014). This finding led to *in vitro* testing where *C. pecorum* was found to survive in tryptophan-free media supplemented with a more diverse pool of tryptophan precursors than *C. trachomatis* could utilize (Islam *et al*. 2018). *C. pecorum* was also found to be completely resistant to IFN-ɣ in human epithelial cells (Islam *et al*. 2018). Translating this genetic difference back to the koala, recent modelling of koala immune parameter data and gene expression analysis has suggested that IFN-ɣ may not play a major role in *C. pecorum* control in koalas (Phillips *et al*.^2019^). This situation is an excellent example of how genome analysis can be the basis of advancing important biological understandings in chlamydial research.

### Prevalence of *C. pecorum* infection and chlamydial disease

**Figure 5.**
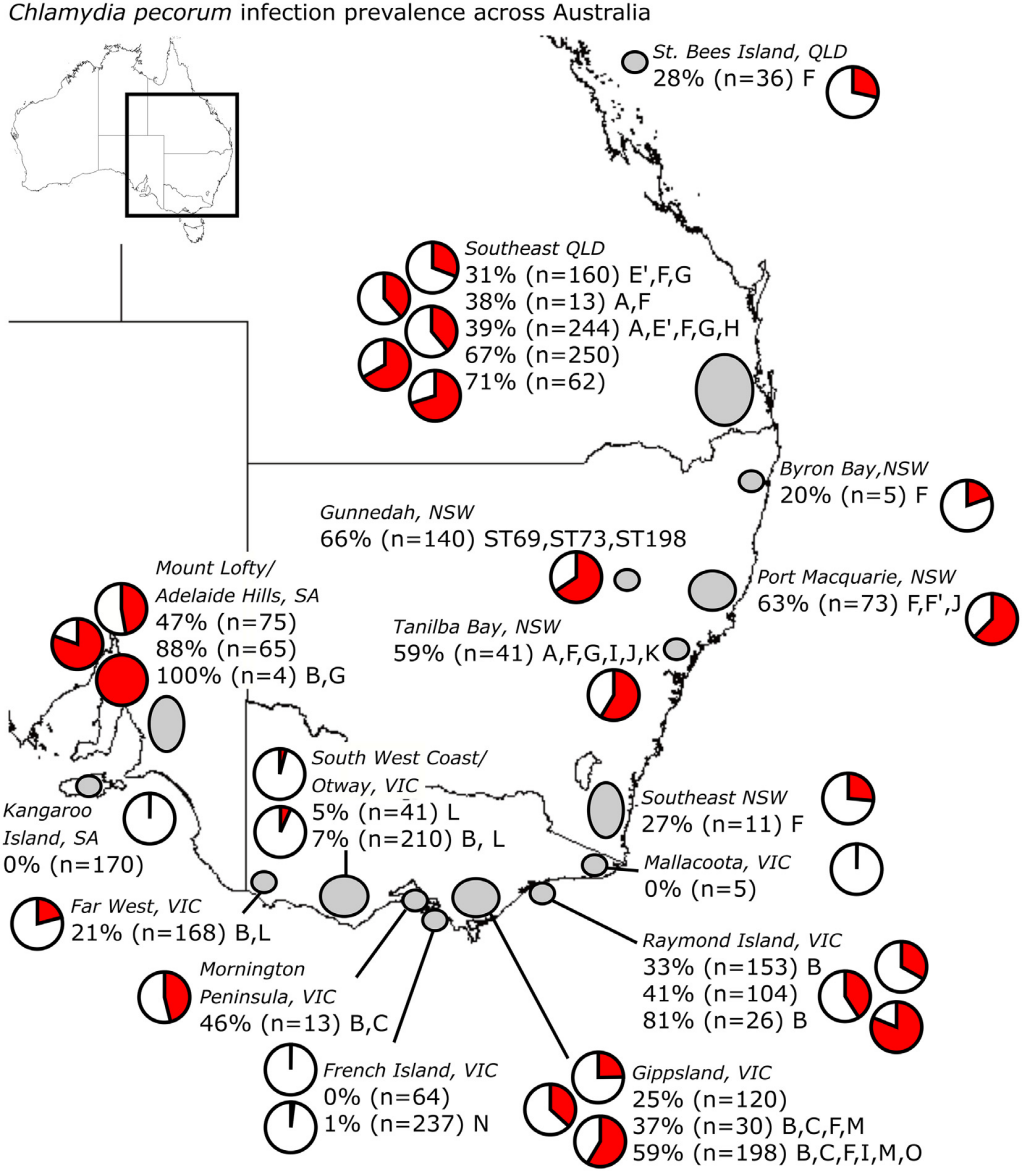
Prevalence of *Chlamydia pecorum* infection reported in studies from 2012 to 2019. Grey ellipticals indicate the mapped area investigated, pie charts and percentages represent the *C. pecorum* positivity reported in ‘n’ number of koalas tested. *C. pecorum ompA* genotype (A-O) or multilocus sequence type (MLST) ST type detected in the study area are given when reported. Details for each study can be found in Table 1.

**Figure 6.**
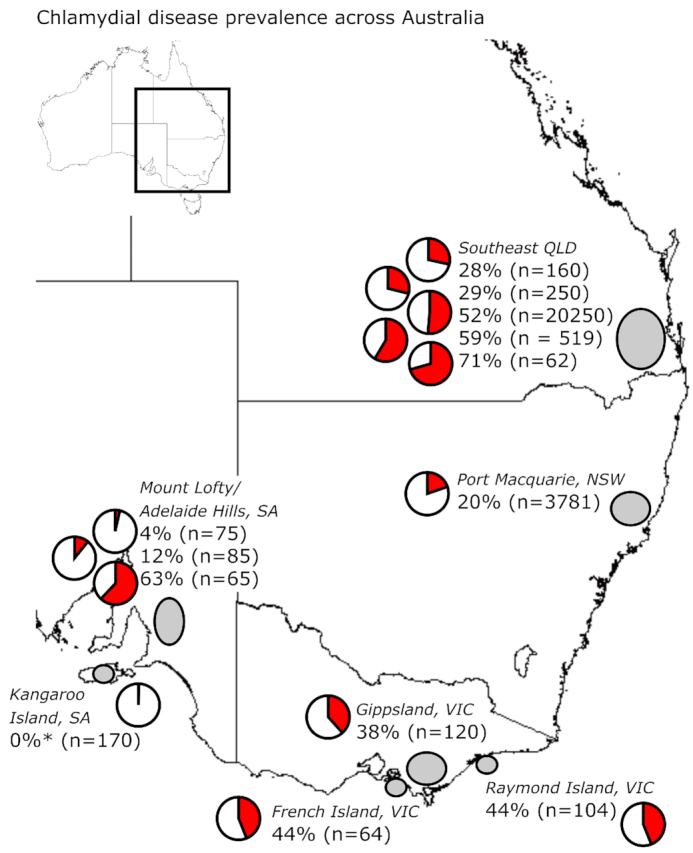
Chlamydial disease prevalence reported in studies from 2012 to 2019. Grey ellipticals indicate the mapped area investigated, pie charts and percentages represent the chlamydial disease reported in ‘n’ number of koalas examined. Details for each study can be found in Table 2. *See clinical disease note in Table 2.

An important area of koala chlamydial research that has received focused attention since 2012 has been the survey and characterization of *C. pecorum* infections and/or chlamydial disease in koalas across Australia (Figs 5 and 6, Tables 1 and 2). A review of *C. pecorum* positivity in koala populations before 2012 found rates from 0% *C. pecorum* detected (from isolated island koala populations) up to 87%–90% positivity in koala populations from QLD and VIC (Polkinghorne, Hanger and Timms 2013). Since that review, there have been 18 additional studies that have investigated some aspect of *C. pecorum* presence and/or chlamydial disease rates in koalas across the range (Table 1A) (Griffith and Higgins 2012; Funnell *et al*. 2013; Griffith *et al*. 2013; Kollipara *et al*. 2013c; Patterson *et al*. 2015; Legione *et al*. 2016a,b; Speight *et al*. 2016; Gonzalez-Astudillo *et al*. 2017; Nyari *et al*. 2017; Speight *et al*. 2018; Wedrowicz *et al*. 2018; Fabijan *et al*. 2019b; Fernandez *et al*. 2019; Gonzalez-Astudillo *et al*. 2019; Hulse *et al*. 2019b; Maher *et al*. 2019; Palmieri *et al*. 2019). These studies have used a range of both established and novel *C. pecorum* detection and genotyping PCR assays (Table 1B) (Devereaux *et al*. 2003; Robertson *et al*. 2009; Griffith 2010; Pantchev *et al*. 2010; Marsh *et al*. 2011; Wan *et al*. 2011; Jelocnik *et al*. 2013; Kollipara *et al*. 2013c; Jelocnik *et al*. 2017; Hulse *et al*. 2018).

**Table 1A.** *Chlamydia pecorum* infection prevalence reported between 2012 and 2019 in Australian koalas.

State	Region	No. Koalas	No. *Chlamydia* Positive (%)	*Chlamydia* genotypes detected	*C. pneumoniae* positive	Reference
QLD	Brisbane	62	509* (71%)	ND	0/677 tissues	Palmieri *et al*. 2019
	Moreton Bay	160	49 (31%)	E’, F, G	ND	Nyari *et al*. 2017
	SEQLD	250	167 (67%)	ND	ND	Hulse *et al*. 2019b
	SEQLD	244	96 (39%)	A, E’, F, G, H	ND	Kollipara *et al*.2013c
	SEQLD	13	5 (38%)	A’, F	ND	Wedrowicz *et al*. 2018
	St Bees	36	10 (28%)	F	ND	Kollipara *et al*.2013c
NSW	Byron Bay	5	1 (20%)	F	ND	Kollipara *et al*.2013c
	Gunnedah	140	93 (66%)	ST69, ST73, ST198	ND	Fernandez *et al*. 2019
	Port Macquarie	73	46 (63%)	F, F’, J	ND	Kollipara *et al*.2013c
	SENSW	11	3 (27%)	F	ND	Wedrowicz *et al*. 2018
	Tanilba Bay	41	24 (59%)	A, F, G, I, J, K	ND	Kollipara *et al*. 2013c
VIC	Cape Otway	41	2 (5%)	L	ND	Wedrowicz *et al*. 2018
	Far West	168	36 (21%)	B	0	Legione *et al*.2016b
	French Island	64	0 (0%)	ND	ND	Patterson *et al*. 2015
	French Island	237	2 (1%)	N	0	Legione *et al*.2016b
	Greater Gippsland	30	11 (37%)	B, C, F, M	0	Legione *et al*.2016b
	Mallacoota	5	0 (0%)	–	ND	Wedrowicz *et al*. 2018
	Mornington Peninsula	13	6 (46%)	B, C	0	Legione *et al*.2016b
	Mt Eccles national park	120	30 (25%)	ND	ND	Patterson *et al*. 2015
	Raymond Island	104	43 (41%)	ND	ND	Patterson *et al*. 2015
	Raymond Island	153	50 (33%)	B	0	Legione *et al*.2016b
	Raymond Island	26	21 (81%)	B	ND	Wedrowicz *et al*. 2018
	South Gippsland	198	117 (59%)	B, C, F, I, M, O	ND	Wedrowicz *et al*. 2018
	South West Coast	210	15 (7%)	B, L	1	Legione *et al*.2016b
SA	Adelaide Hills	4	4 (100%)	B, G	ND	Kollipara *et al*.2013c
	Kangaroo Island	170	0 (0%)	–	ND	Fabijan *et al*.2019b
	Mount Lofty	75	35 (47%)	ND	ND	Fabijan *et al*.2019b
	Mount Lofty (+ Eyre Peninsula)	65	57 (88%)	ND	ND	Speight *et al*. 2016

*677 tissues tested.

**Table 1B.** Tests used to determine *C. pecorum* infections prevalence.

Study reference	Gene target and primers for *C. pecorum* test	Reference for *C. pecorum* test	Gene target and primers for genotyping	Reference for genotyping
Kollipara *et al*. 2013c	16S gene - RT-Pec.spF/R	Marsh *et al*. 2011	ompA - ompA-F/R	Kollipara *et al*. 2013c
Patterson *et al*. 2015	16S gene - 16SG F/R	Robertson *et al*. 2009	ND	ND
Legione *et al*.2016b	16S gene - 16SG F/R	Robertson *et al*. 2009	ompA - ompA-F/R	Kollipara *et al*. 2013c
Speight *et al*. 2016	16S gene - 16Sf/16Sr	Wan *et al*. 2011	ND	ND
Nyari *et al*. 2017	16S gene - RT-Pec.spF/R	Marsh *et al*. 2011	ompA - CpeNTVD3/4	Devereaux *et al*. 2003
Wedrowicz *et al*. 2018	ompA - CppecOMP1-F/R/S	Pantchev *et al*. 2010	ompA - ompA-F/R	Kollipara *et al*. 2013c
Hulse *et al*. 2019	ompB - CpecOmpB-F/R	Hulse *et al*. 2018	ND	ND
Palmieri *et al*. 2019	ompB - CpecOmpB-F/R	Hulse *et al*. 2018	ND	ND
Fabijan *et al*. 2019b	HP gene - B3/F3	Jelocnik *et al*. 2017	ND	ND
Fernandez *et al*. 2019	ompB - F/R	Griffith 2010	MLST - gatA, oppA_3, hflX, gidA, enoA, hemN, fumC	Jelocnik *et al*. 2013

On one end of the spectrum, when more than five koalas have been tested in an area, only Kangaroo Island, South Australia (SA) has remained apparently *C. pecorum*-free (Figs 5 and 6) (Fabijan *et al*. 2019b). In this population, while no overt cases of chlamydial disease were observed, 7% of examined koalas presented with very mild ocular clinical scores (Table   2) (Fabijan *et al*. 2019b). It is well established that koalas can have chlamydial disease signs with no detectable *C. pecorum* organisms present (Wan *et al*. 2011; Nyari *et al*. 2017; Legione *et al*. 2018; Quigley *et al*. 2018a), so whether this disparity on Kangaroo Island represents a situation where *C. pecorum* organisms were present at low levels and were naturally cleared before testing or whether clinical signs were due to another aetiological agent remains an open question. French Island, VIC is another island koala population that had historically tested *C. pecorum*-free, but with signs of chlamydial disease (44% of koalas observed with “wet bottom”) (Tables 1 and 2) (Patterson *et al*. 2015). Subsequent extended survey and analysis has since found these koalas to be infected with a *C. pecorum* strain more genetically similar to known livestock strains compared to koalas strains, now designated *ompA* genotype N (Legione *et al*.2016a,b). As island koala populations continue to be examined in greater depth and detail, hope fades that any wild koala population will continue to test *C. pecorum*-free.

**Table 2.** Chlamydial disease prevalence reported between 2012 and 2019 in Australian koalas.

State	Region	No. Koalas	No. chlamydial disease (%)	Reference	Notes
QLD	Brisbane	62	44 (71%)	Palmieri *et al*. 2019	Males only, Moggill Koala Hospital
	Moreton Bay	160	44 (28%)	Nyari *et al*. 2017	
	SEQLD	250	65 (29%)	Hulse *et al*. 2019	Males only
	SEQLD	20250**	21619 (52%)	Gonzalez-Astudillo *et al*. 2017	Moggill Koala Hospital records from 1997–2013
	SEQLD	519	304 (59%)	Gonzalez-Astudillo *et al*. 2019	Moggill Koala Hospital, Currumbin Wildlife Sanctuary Hospital, and Australia Zoo Wildlife Hospital, records from 2013–2016
NSW	Port Macquarie	3781***	771 (20%)	Griffith *et al*. 2013	Port Macquarie koala hospital records 1975–2004
VIC	French Island	64	28 (44%)	Patterson *et al*. 2015	Disease was observed wet bottom
	Mt Eccles national park	120	46 (38%)	Patterson *et al*. 2015	Disease was observed wet bottom
	Raymond Island	104	45 (44%)	Patterson *et al*. 2015	Disease was observed wet bottom
SA	Adelaide	85	10 (12%)	Speight *et al*. 2018	Survey of deceased koalas at Veterinary School
	Kangaroo Island	170	0 (0%)	Fabijan *et al*. 2019a	12 koalas (7%) had ocular clinical scores of 1 (very mild)
	Mount Lofty	75	3 (4%)	Fabijan *et al*. 2019a	All three cases were severe disease
	Mount Lofty (+ Eyre Peninsula)	65	41 (63%)	Speight *et al*. 2016	

**41 606 aetiologies determined from 20 250 koalas from Moggill koala hospital records from 1997 to 2013, with *Chlamydia* infection being the most common; ***Port Macquarie koala hospital records 1975–2004, with chlamydiosis being the second most common aetiology.

Across mainland Australia, where more than five koalas from an area have been tested, *C. pecorum* infection rates have ranged from 21%–88% (Fig. 5, Table 1). This range is seen not only across the country, but also within regions where multiple studies have been conducted over different years (southeast QLD: 31%–71%; Raymond Island, VIC: 33%–81%; Gippsland, VIC: 25%–59%; Mount Lofty, SA: 47%–88%) (Fig. 5). This suggests that *C. pecorum* infection rates are dynamic within a koala population, most likely influenced by a range of host and environmental pressures. As noted in the *C. pecorum ompA* genotyping discussion, there is a trend for some *C. pecorum ompA* genotypes, such as genotypes B, C, L and M, to be found only in southern (VIC and SA) koalas while other strains, like genotypes A, E’ and F’ are present only in northern (QLD and NSW) koalas (Fig. 5, Table 1). However, there does not appear to be any pattern between *ompA* genetic differences and *C. pecorum* infection rates in koala populations at the locations surveyed to date.

Studies have also focused on documenting clinical chlamydial disease in koala populations across Australia. These studies generally reflect *C. pecorum* infection prevalence, with chlamydial disease prevalence ranging from 4%–71% across the country (Fig. 6 and Table 2). Included in these data are three surveys of koala hospital records: (i) Moggill Koala Hospital (in Brisbane, QLD) reporting a chlamydial disease prevalence of 52% (n = 20 250) of admitted koalas between 1997 and 2013 (Gonzalez-Astudillo *et al*. 2017), (ii) Moggill Koala Hospital, Currumbin Wildlife Sanctuary Hospital and Australia Zoo Wildlife Hospital (all southeast QLD) reporting a combined chlamydial disease prevalence of 59% (n = 519) in necropsied koalas between 2013 and 2016 (Gonzalez-Astudillo *et al*. 2019) and (iii) Port Macquarie Koala Hospital (Port Macquarie, NSW) reporting a chlamydial disease prevalence of 20% (n = 3781) of admitted koalas between 1975 and 2004 (Griffith *et al*. 2013). While koala hospital surveys offer useful insights into disease prevalence in an area over time, it should be remembered these types of surveys are inherently biased towards animals that show overt signs of distress and are brought into care. Less externally apparent signs of chlamydial disease may not be recognized in the community, leading to fewer of these koalas being brought into hospital care and recorded in prevalence measures. Attempting to address this bias, one study that focused on characterizing chlamydial disease signs not readily observable found 75% (n = 62) of koalas examined from Port Macquarie Koala Hospital without “wet bottom” or conjunctivitis had lesions attributable to chlamydiosis only observable by ultrasonography and/or at necropsy (Marschner *et al*. 2014). Another study found that 51% (n = 65) of koalas from the Mount Lofty Ranges and Eyre Peninsula, SA had chlamydial lesions that were only detectable microscopically or by histopathology during necropsy (Speight *et al*. 2016). These studies, as well as others (Patterson *et al*. 2015; Nyari *et al*. 2017), highlight that only a limited spectrum of chlamydial disease clinical signs can be easily visualized without specialist equipment or internal examination and suggest that the full impact and burden of chlamydial disease may be underrepresented when only classical visual assessment methods are used.

Finally, the situation where koalas present with the classic signs of chlamydial disease, but with no detectable *C. pecorum*, should also be acknowledged. Cross-sectional studies that have evaluated both chlamydial infection and disease in the same animal routinely report individuals with disease signs clinically attributable to chlamydial disease but without *C. pecorum* being detected by molecular testing of samples (Patterson *et al*. 2015; Nyari *et al*. 2017; Quigley *et al*. 2018a). The definitive reason for these discrepancies is currently unknown, but theories to account for these differences include an alternative pathogen(s) in koalas causing the same clinical signs (Patterson *et al*. 2015), variation in organism shedding during the course of disease or the resolution of the *C. pecorum* infection before/without the resolution of clinical signs (Nyari *et al*. 2017).

### Chlamydial disease and host response

Chlamydiosis is a well-characterized disease in koalas. In the last 10 years, two areas of research have made notable advancements in our understanding of chlamydial disease in koalas: disease progression over time and the koala immune response to infection. Based on the recognition that koala chlamydiosis research was lacking population-level, long-term disease studies (Grogan *et al*. 2017; McCallum *et al*. 2018), researchers followed koala populations over several years to investigate more complex questions related to disease progression. In addition, the release of the first complete koala genome (Johnson *et al*. 2018), as well as specialized koala transcriptome datasets (Hobbs *et al*. 2014; Morris *et al*. 2014; Abts, Ivy and DeWoody 2015; Morris *et al*. 2016), have allowed for koala-specific immune targets to be specifically investigated. Together, these advances have provided the foundation to delve more deeply into chlamydial disease progression in koalas.

Unlike *C. trachomatis* infections in people, which are most often asymptomatic (Ziklo *et al*. 2016a), longitudinal monitoring of a wild koala population in southeast QLD found that 66% (n = 38) of koalas with *C. pecorum* infections progressed to clinical disease (Robbins *et al*. 2019). This observation addressed a long-standing question from cross-sectional studies where koalas are routinely found to be *C. pecorum* positive by PCR but with no apparent clinical signs (Nyari *et al*. 2017; Quigley *et al*. 2018a) – will these koalas resolve the asymptomatic infection without intervention or will they progress to disease? This longitudinal study suggests that 2/3 of koalas with progress to disease while 1/3 with recover without intervention (Robbins *et al*. 2019). Disease progression at the urogenital site has been found to be associated with urogenital *C. pecorum* load (Robbins *et al*. 2019). Urogenital *C. pecorum* load was also found to be an important factor in disease progression in several modeling studies (Quigley *et al*. 2018b). Additionally, antibiotic treatment of chlamydial disease was shown to provide only short-term protection, with treated koalas acquiring new *C. pecorum* infections and disease signs within six months of previous disease resolution (Robbins *et al*. 2019). Taken together, these longitudinal data indicate that *C. pecorum* infection appears to progress to disease regularly and recovery from disease after treatment seems to offer the koala little protection against future infection and disease.

Cytokines play a critical role in the immune system, regulating and directing the immune response to invading pathogens. Assays targeting koala cytokines, including the pro-inflammatory cytokine Tumour Necrosis Factor alpha (TNF-α), pro-inflammatory Th1 response cytokine IFN-ɣ, pro-inflammatory Th17 response cytokine Interleukin 17A (IL-17A), Th2 response cytokine Interleukin 4 (IL-4) and the anti-inflammatory Th2 response cytokine Interleukin 10 (IL-10), have all been developed (Mathew *et al*. 2013a,b; Maher *et al*. 2014; Mathew *et al*. 2014). In addition, assays for koala Interleukin 6 (IL-6), known to be expressed by epithelial cells during chlamydial infection (Cunningham *et al*. 2013), as well as koala-specific CD4 and CD8β markers, have also been developed (Maher *et al*. 2014). Comparing peripheral blood mononuclear cells (PBMCs) from koalas with current chlamydial disease, asymptomatic chlamydial infection and no chlamydial infection/disease determined that koalas with active disease had significantly higher expression of TNF-α, INF-ɣ and IL-10 (Mathew *et al*. 2013a,b). These results were further supported by a subsequent study that detected significantly higher expression of IL-17A and modest increases in expression of TNF-α and IFN-ɣ in currently chlamydial diseased koalas (Mathew *et al*. 2014). However, cytokine involvement in the immune response to *C. pecorum*, particularly in relation to the IFN-γ, is still an unclear area in koala chlamydial disease research. Investigation of IFN-γ during chlamydial disease has expanded to include both targeted gene studies (Mathew *et al*. 2013b) and total RNA expression within the cell (Phillips *et al*. 2019), with the importance of IFN-γ appearing different between the different study methods. Given the complexity of intracellular pathways and networks, it is not surprising that discrepancies will arise between studies. Future research in this area will be necessary to clarify the roles of all important cytokines, not just IFN-γ, for the safe implementation of treatment options, like vaccination, to koalas.

### *Chlamydia pecorum* transmission

*Chlamydia* are well-established as sexually transmitted pathogens and sexual contact appears to be the primary transmission route of *C. pecorum* between koalas (Polkinghorne, Hanger and Timms 2013). However, investigations have expanded our awareness of non-sexual *C. pecorum* transmission, primarily between mothers (dams) and offspring (joeys). Two recent studies focused on joeys that were either still dependent (with their dams, less than one year old) or still sexually immature (between 9 and 13 months old) and both studies found a 27% *C. pecorum* positivity in the joeys ((n = 15) (Nyari *et al*. 2017); (n = 11) (Russell *et al*. 2018)). The dependent joey study (Russell *et al*. 2018) was based on koalas in care, so while dam-joey transmission was suspected as the primary route of *C. pecorum* transmission to the joeys, handling of dams and joeys by the same animal handler could not be completely ruled out. Alternatively, the sexually immature joey study (Nyari *et al*. 2017) was conducted from a monitored wild koala population where routes of *C. pecorum* transmission to joeys other than dam-to-joey were unlikely. These studies reveal that koalas less than one year old are acquiring chlamydial infections through non-sexual transmission routes and future management strategies will need to take this into account.

Another *C. pecorum* transmission concern is when koalas are translocated between different geographical sites with different chlamydial characteristics (Waugh *et al*.2016a). A translocation study was carried out in VIC where 30 *C. pecorum* negative (also *Chlamydia*-antibody negative) koalas were moved from French Island, VIC (where *C. pecorum* had not been detected up to this point) to forests near Ballarat, VIC (on the mainland) (Santamaria and Schlagloth 2016). After the first breeding season on the mainland (six months post-translocation), 25 of the translocated koalas were examined to find 48% (12/25) were now *Chlamydia*-antibody positive, with no detectable *C. pecorum* infections and six live joeys in the group. After the second breeding season (19 months post-translocation), 17 of the translocated koalas were examined to find 94% (16/17) were now *Chlamydia*-antibody positive, with 56% (9/16 (one koala was not swabbed for culture)) now having a chlamydial infection (seven with *C. pecorum*, one with both *C. pecorum* and *C. pneumoniae* and one with *C. pneumoniae*) and only one live joey in the group (Santamaria and Schlagloth 2016). The study concluded that moving koalas without carefully determining the chlamydial disease status of both the translocating koalas and the translocation site should be regarded as detrimental to the animals and translocation should only be undertaken with the greatest possible care and monitoring (Santamaria and Schlagloth 2016).

### Detection of *C. pecorum*

As our understanding of both chlamydial infection and disease has progressed since 2012, so has the development of tools to detect *C. pecorum* and chlamydial disease in koalas. Progress has been made in understanding which samples should be tested and how both molecular and non-molecular techniques can contribute to pathogen and disease detection. Recognizing that non-invasive koala sampling, such as scat detection and testing, can be a low cost, non-disruptive survey method, effort has been put into testing whether *C. pecorum* detection in scat is comparable to direct urogenital swab sampling by quantitative PCR (qPCR) (Wedrowicz *et al*. 2016). In a small study, testing found a high level of concordance (83%; 5/6) between paired scat and urogenital swab samples, although the same *C. pecorum ompA* genotype was not always found between samples from the same koala (Wedrowicz *et al*. 2016). Perhaps unsurprisingly, *C. pecorum* copy numbers were consistently higher from urogenital swab samples compared to paired scat samples (Wedrowicz *et al*. 2016). Overall, this data suggests that non-invasive koala sampling has lower *C. pecorum* sensitivity compared to samples taken directly from a koala. This difference should be kept in mind when comparing prevalence estimates generated from different sampling methods.

Molecular tests for *C. pecorum* are the gold standard for detection, with established assays targeting the *C. pecorum* 16S rRNA gene (Marsh *et al*. 2011; Wan *et al*. 2011), *ompA* gene (Marsh *et al*. 2011; Higgins *et al*. 2012) and the CpecG_0573 (hypothetical protein (HP)) gene (Fabijan *et al*. 2019b; Robbins *et al*. 2019). Advances to these molecular tests have come in the forms of multiplexing and rapid point-of-care (POC) adaptations. Increasing the throughput of testing with multiplexing, real-time PCR assays have been developed to detect either the combination of the koala beta-actin gene and genus level *Chlamydia*, *Mycoplasma* and *Ureaplasma* DNA or species-specific *C. pecorum*, *C. pneumoniae* and *Bordetella bronchiseptica* (Hulse *et al*. 2018). Further accelerating detection from qPCR to POC diagnostics, two loop‐mediated isothermal amplification (LAMP) assays have recently been developed for *C. pecorum* detection. The first LAMP assay, released in 2017, targets a hypothetical protein gene, CpecG_0573, determined to be unique to *C. pecorum* based on genomic analysis (Jelocnik *et al*. 2017), while the second assay, released in 2019, targets the *mreC* gene (coding for a cell shape determining protein) from *C. pecorum* (Hulse *et al*. 2019a). Both assays report no cross-reactivity with a range of non-target organisms and rapid sample preparation protocols for minimal sample preparation, making them both candidates for POC diagnostics in the field or veterinary clinic (Jelocnik *et al*. 2017; Hulse *et al*.2019a).

Non-molecular tests have also been evaluated in recent years for *C. pecorum* infection or disease detection. For a time, an enzyme immunoassay, Clearview, was available for *C. pecorum* POC testing. Comparison to qPCR found that Clearview was 93% specific for *C. pecorum*, but only 43% sensitive (needing +400 copies of *C. pecorum* genomic DNA per test for detection) (Hanger *et al*. 2013). However, since this evaluation, the Clearview test is no longer available commercially. Detecting chlamydial disease, either in cases where overt clinical signs are absent or from koala samples like scat, has been another area that has received attention in recent years. A review was conducted at the Port Macquarie Koala Hospital to determine if ultrasonography was an accurate method to detect urogenital tract structural disease in koalas (Marschner *et al*. 2014). By comparing ultrasonography observations to paired necropsy results, the study found strong positive agreement in results from 86% of kidneys, 90% of bladders and 93% of ovarian bursal cysts, indicating ultrasonography was an effective diagnostic tool for assessing structural damage caused by chlamydial disease in koalas (Marschner *et al*. 2014). Another study sought to determine if koala detection dogs could recognize koala scats from actively diseased koalas compared to healthy koalas (Cristescu *et al*. 2019). While sample sizes were limited, the detection dogs could distinguish scat from clinically diseased koalas (n = 5) verses koalas that showed no clinical signs (n = 13), most likely recognizing a volatile organic compound difference in the scats (Cristescu *et al*. 2019). These alternative infection and disease determining techniques continue to broaden the toolkit available to koala disease researchers for better diagnosis and monitoring of chlamydial infection and disease in koala populations.

### Treatments for *C. pecorum* – antibiotics and vaccines

Because the koala gastrointestinal tract is full of specialized bacteria needed for the digestion of eucalyptus leaves, koalas are especially sensitive to caeco-colic dysbiosis/typhlocolitis syndrome from antibiotic-induced microbial dysbiosis (Gillett and Hanger 2019). This disruption to the koala gut microbiome can be fatal, making antibiotic use to treat chlamydial disease in koalas a delicate balancing act. Traditional *C. pecorum* antibiotic treatments include the use of chloramphenicol and enrofloxacin, with enrofloxacin treatment failure a known issue (Polkinghorne, Hanger and Timms 2013). More recent research into the minimum inhibitory concentration (MIC) needed to control *C. pecorum* isolates from koalas revealed that the dosage of enrofloxacin needed to kill *C. pecorum* was above the conventional dose rate, possibly explaining these previous treatment failures (Black, Higgins and Govendir 2015). This has made chloramphenicol the treatment of choice for chlamydial disease in koalas. Chloramphenicol has traditionally been recommended at a dosage of 60 mg/kg for 45 days and even at this dosage, severe urogenital disease did not respond well (Govendir *et al*. 2012). Recent re-evaluation of this treatment regimen found that after koalas with a poor prognosis were removed from treatment on animal welfare grounds, a 60 mg/kg dosage of chloramphenicol for only 14 to 28 days had a successful treatment rate of 95% (Robbins *et al*. 2018). However, despite the success of chloramphenicol for koala chlamydial disease treatment, significant side effects, including bone marrow depression and fatal caeco-colic dysbiosis, are still seen with prolonged use (Govendir *et al*. 2012). Additionally, the supply of chloramphenicol base (the most effective form of the antibiotic (Black *et al*. 2013)) is not secure, with a commercial suspension of this antibiotic's production in 2013–2014. This shortage lead to investigations into alternative antibiotics suitable for koala use.

Alternative antibiotics such as doxycycline, florfenicol and penicillin G have all been considered for chlamydial disease treatment in koalas. Doxycycline given at 5 mg/kg diluted 50:50 in sterile saline once a week for four weeks has been found to reverse the signs of clinical cystitis, eliminate “wet bottom” and clear *C. pecorum* infection in a small group of koalas (n = 3) (Phillips *et al*. 2019). Florfenicol treatment at dosages tolerable in the field had limited success, with only 26% (n = 5) of treated koalas resolving their clinical signs and being released without further treatment, with another 32% (n = 6) requiring additional treatment with chloramphenicol to resolve their disease signs and the remaining animals (36%, n = 7) failing to clinically improve (Budd *et al*. 2017). Finally, penicillin G did not make it out of *in vitro* testing, where cell culture testing found this antibiotic induced a chlamydial stress response (into persistence) in *C. pecorum* and was not bactericidal (Leonard, Dewez and Borel 2016). Given these results, there is still a great need for alternatives to antibiotic treatment for chlamydial disease management in koalas.

The quest for a koala chlamydial vaccine has recently been reviewed (Phillips, Quigley and Timms 2019). Modelling has been done to predict the effect a chlamydial vaccine could have on declining koala populations in southeast QLD (an area endemic with *Chlamydia*) (Craig *et al*. 2014). With other koala threats remaining constant, modeling predicted that a vaccine with 75% protective efficacy, covering around 10% of the koala population each year and targeting young female koalas could reverse current population declines in five to six years (Craig *et al*. 2014). Several chlamydial vaccine formulations have been tested, including different adjuvants and *C. pecorum* target proteins, to determine which combinations produced the strongest humoral and cellular immune responses and offered the best disease protection (Kollipara *et al*. 2012; Kollipara *et al*.2013a,b; Khan *et al*. 2014; Waugh *et al*. 2015; Khan *et al*. 2016a,b; Waugh *et al*. 2016c,d; Desclozeaux *et al*. 2017; Nyari *et al*. 2018, 2019). Presently, a recombinant MOMP *C. pecorum* vaccine has shown the most promising protection overall, as well as having the therapeutic potential to replace antibiotics for mild ocular disease (Desclozeaux *et al*. 2017; Lizárraga, Carver and Timms 2019; Nyari *et al*. 2019). Further efforts to refine the recombinant MOMP vaccine into a peptide-based vaccine have shown promising results (Nyari *et al*. 2018), but more work on this formulation is still needed. In addition, long-term monitoring of vaccinated and control koalas (n = 106) has revealed that chlamydial vaccination also had positive effects on koala lifespan, adding a median of 3.5 years to vaccinated koala's lives (lifespan of 12.25 years for vaccinated koalas verses 8.8 years for unvaccinated koalas) (Hernandez-Sanchez *et al*. 2015). It was believed that, in additional to avoiding chronic illness, this generalized longer life may have been due to cross-reactive adaptive immune responses (heterologous immunity) together with epigenetic reprogramming of the innate immune system (trained innate immunity) (Hernandez-Sanchez *et al*. 2015). Finally, the benefits of chlamydial vaccination have not only been realized in the individual animals vaccinated, but also potentially in their future offspring. Preliminary evidence from five dam-joey pairs found that the joeys of vaccinated dams (n = 3) remained chlamydial infection and disease free as they became independent from their mothers while joeys from unvaccinated dams (n = 2) developed either chlamydial infection or both infection and disease as they became independent (Russell *et al*. 2018). Although passive immunity from mother to offspring is a well-recognized immunological process, further research into its place in koala chlamydial vaccination is needed before conclusive statements can be made. However, collectively, the evidence that chlamydial vaccination has a positive impact on koalas is continuing to grow with each study.

## KOALA RETROVIRUS (KoRV)

**Figure 7.**
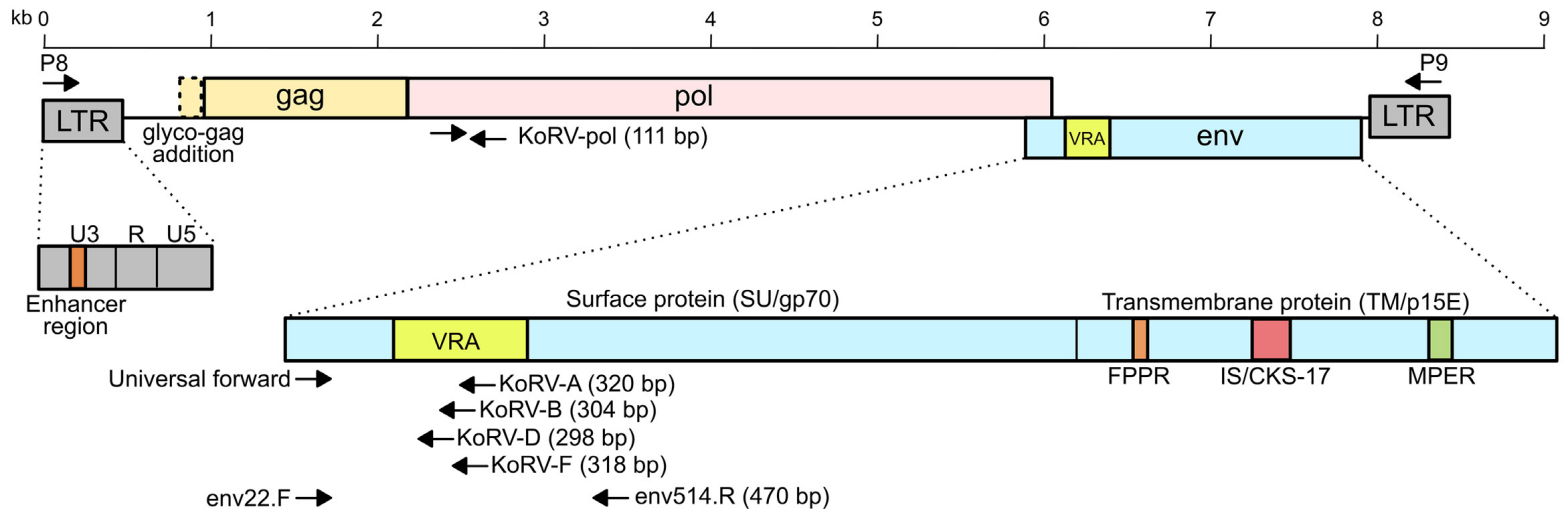
Schematic of koala retrovirus (KoRV) with discussed features highlighted. LTR - Long terminal repeats, gag - the group-specific antigen gene, pol - protease-polymerase gene, env - envelope gene, VRA – variable region A (receptor binding domain), FPPR – fusion peptide-proximal region, IS/CKS-17 – immunosuppressive domain, MPER – membrane-proximal external region. Arrows indicate PCR primer binding sites for common assays.

As early as 1988, researchers noted by electron microscopy that koalas with leukemia had detectable *Gammaretrovirus*-like (type C retrovirus) particles budding from their cancerous cells (Canfield, Sabine and Love 1988). However, it took until 2000 for a retrovirus in koalas to be fully recognized, sequenced and named koala retrovirus (KoRV) (Fig. 7) (Hanger *et al*. 2000). At this early stage, it was recognized that KoRV shared remarkable homology with gibbon ape leukemia virus (GALV) and had a proviral integration pattern consistent with an endogenous retrovirus (a virus that has incorporated into germline cells and is transmitted from parent to offspring in the chromosomal DNA) (Hanger *et al*. 2000). Continued investigation confirmed that KoRV was indeed endogenously incorporated into the koala genome in northern koalas (from QLD and NSW), while still believed to be exogenous (transmitted horizontally between koalas through infection) in the south (VIC and SA) (Tarlinton *et al*. 2005; Tarlinton, Meers and Young 2006). In addition, KoRV was found to produce intact viral particles in infected animals, indicating that the virus appeared to be both endogenous and transmissible (Tarlinton *et al*. 2005; Tarlinton, Meers and Young 2006). Importantly for koala health, it was also determined that increased plasma levels of KoRV RNA could be associated with koalas who developed leukemia and lymphoma, as well as clinical chlamydiosis (Tarlinton *et al*. 2005). By 2012, KoRV was clearly recognized as a pathogen of koalas. However, focused study of this virus since 2012 has revealed a complex diversity and epidemiology. As our understanding of KoRV has evolved over the last eight years, several reviews have tried to capture the current state of knowledge about this virus (Tarlinton 2012; Denner and Young 2013; Xu and Eiden 2015; Denner 2016; Kinney and Pye 2016; Greenwood *et al*. 2018; Higgins and Maher 2019). However, research into KoRV has progressed as fast as reviews could be written, with new discoveries routinely being made in understanding where KoRV came from, what it is doing and how we may be able to intervene to help the koala.

### Origin of KoRV in koalas

KoRV was first recognized as a close relative of GALV. Given that GALV appeared as a pathogenic exogenous virus in a captive gibbon research facility in Thailand and has never been detected in gibbons other than within this one spill-over event (Brown and Tarlinton 2017) and koalas are native to Australia, there has been an active interest in identifying the reservoir host of the precursor *Gammaretrovirus* that became KoRV. The first reasonable candidate emerged from the native Australian rodent, the grassland melomys, *Melomys burtoni*. A screen of 42 vertebrate species in Australia (including rodents and bats) identified a novel *Gammaretrovirus* with 83% identity to KoRV and 93% identity to GALV in *M. burtoni*, leading to this virus being designated Melomys burtoni retrovirus (MbRV) (Simmons *et al*. 2014). Despite the overlap in geographical ranges between melomys and koalas, MbRV appeared to be a defective endogenous retrovirus, not producing any detectable viral RNA or virus particles, reducing its likelihood of being the source of KoRV (Simmons *et al*. 2014). An extended *Gammaretrovirus* search in Southeast Asia detected another virus in *M. burtoni* from Indonesia, distinct from MbRV, which was named *Melomys* woolly monkey virus (MelWMV) (Alfano *et al*. 2016). However, MelWMV also appeared to be a defective endogenous retrovirus (missing major gene regions), so again, not a likely source for GALV and KoRV (Alfano *et al*. 2016). Most recently, gammaretroviruses have been identified from black flying-foxes (*Pteropus alecto*) from QLD and were designated flying-fox retrovirus (FFRV) (McMichael *et al*. 2019) and Hervey pteropid gammaretrovirus (HPG) (Hayward *et al*. 2020). Analysis of the full-length, intact FFRV and HPG sequences found that these gammaretroviruses are distinct from other known bat viruses and instead phylogenetically grouped quite closely with KoRV and GALV (McMichael *et al*. 2019; Hayward *et al*. 2020). Bats have been hypothesized as sources of novel retroviruses before, with a defective retrovirus of some similarity to KoRV having been detected in the microbat *Megaderma lyra* (*Megaderma lyra* retrovirus, MIRV) in China previously (Cui *et al*. 2012). Bats are well-known for their ability to travel long distances and the black flying-fox has a known geographic range that includes Australia, Papua New Guinea and Indonesia (Roberts *et al*. 2017). So, as the search for the reservoir host of the *Gammaretrovirus* that spilled over into koalas to become KoRV continues, this research continues to expand our knowledge of rodent and bat gammaretroviruses and the possible interactions between these animals and koalas.

### Advances in the genetic understanding of KoRV

**Figure 8.**
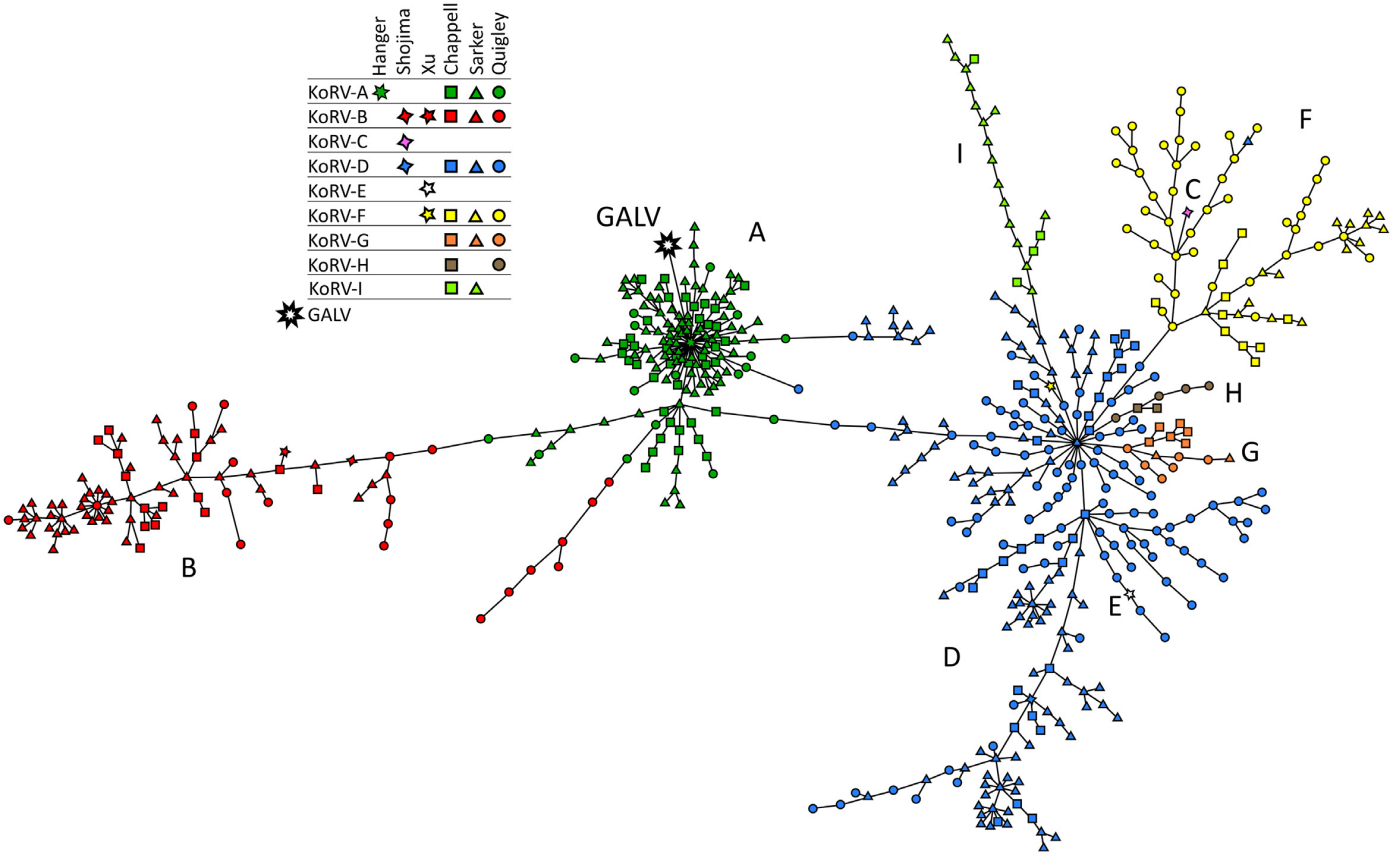
KoRV diversity detected from koalas, visualized by minimum spanning tree (PHYLOViZ, http://online.phyloviz.net/index). Reference strains including Hanger *et al*. 2000 (original KoRV/KoRV-A), Shojima *et al*. 2013 (KoRV-B/J = OJ-4, 11–4; KoRV-C = OJ-4, 11–2; KoRV-D = OJ-4, 11-1), Xu *et al*. 2013 (KoRV-B) and Xu *et al*. 2015 (KoRV-E and -F), Chappell *et al*. 2017; Sarker *et al*. 2019 and Quigley 2020 (accession numbers MN931399-MN931590).

Some of the most exciting and technically advanced work that has been done with KoRV in recent years relates to our understanding of this virus on a genetic level. In 2012, KoRV was considered a single virus while, by 2020, there are now nine subtypes recognized (Fig. 8). Beyond investigating the diversity of this virus, work has also focused on understanding basic viral properties, understanding viral integration into the koala genome, expanding our knowledge of defective KoRV variations, and learning what other endogenous retroviruses (ERVs) in the koala that KoRV may interact with. Collectively, this body of work has not only advanced KoRV understanding but has added important information to our knowledge of retrovirology in general.

Gammaretroviruses are categorized based on the cellular receptors they use for infection. The original KoRV isolate (now known as KoRV-A) was shown to use the same receptor for cell entry as GALV – the sodium-dependent phosphate transporter membrane protein PiT1 (Oliveira, Farrell and Eiden 2006). Next, within months of each other in 2013, two reports were published on a new KoRV subtype that both used a different cell receptor, the thiamine transporter 1 (THTR1), to enter cells (Shojima *et al*. 2013b; Xu *et al*. 2013). The first report came from captive koalas at Los Angeles Zoo suffering from lymphoma and this KoRV strain was designated KoRV-B (Xu *et al*. 2013). The second report came from QLD koalas reared at the Kobe Municipal Oji Zoo in Hyogo, Japan and the KoRV strain was designated KoRV-J (Shojima *et al*.2013b). Very quickly, phylogenetic analysis determined that KoRV-B and KoRV-J represented the same subtype and were grouped together with the designation KoRV-B (Fig. 8) (Shimode *et al*. 2014). Furthermore, phylogenetic analysis indicated that additional KoRV isolates from the Kobe Zoo study contained enough differences in their putative receptor binding domains (within the variable region A [VRA] of their envelope genes) that they should be designated as novel subtypes – KoRV-C and KoRV-D (Fig. 8) (Shimode *et al*. 2014). In the same year, KoRV-B was also detected for the first time in a wild Australian koala (a female from the Port Macquarie Koala Hospital (NSW)) (Hobbs *et al*. 2014). Additional studies followed from koalas at the San Diego and Los Angeles Zoos that isolated unique KoRV variants that also used different cell receptors from PiT1 and THTR1 and these KoRV subtypes were designated KoRV-E and KoRV-F (Fig. 8) (Xu *et al*. 2015). Finally, a survey study of wild southeast QLD koalas targeting the VRA region of the KoRV envelope gene found additional receptor binding domain diversity and established the subtypes KoRV-G, KoRV-H and KoRV-I (Fig. 8) (Chappell *et al*. 2017).

Based on the phylogenetic diversity of KoRV envelope VRA regions, KoRV currently segregates into 3 major clades representing KoRV-A (PiT1 receptor), KoRV-B (THTR1 receptor) and KoRV-C-I (unknown receptor(s)), with these groupings robustly reproduced in larger survey studies that have encompassed wild koalas from across Australia (QLD and SA – (Sarker *et al*. 2019); QLD, NSW and VIC – Quigley (accession numbers MN931399-MN931590) ) (Fig.   8). As more KoRV sequences have been characterized, it has become clear that the original KoRV-C sequence (Shojima *et al*. 2013b) now belongs to the characterized KoRV-D or -F subtype and the original KoRV-E and KoRV-F sequences (Xu *et al*. 2015) now belong to the large and diverse KoRV-D subtype (Fig. 8). In fact, given the diversity of the VRA region within the KoRV-D subtype and the uncertainty of KoRV-C to KoRV-I cell receptor use, support for the use of all the subtype designations (especially KoRV-G and KoRV-H) is variable (Fig. 8) (Sarker *et al*. 2019). Regardless, despite nine subtypes of KoRV officially being described, there are generally three (KoRV-A, -B, -D) to seven (KoRV-A, -B, -D, -F, -G, -H, -I) subtypes routinely reported (Chappell *et al*. 2017; Quigley *et al*. 2019; Sarker *et al*. 2019) (Table 3A).

**Table 3A.** KoRV infection prevalence reported between 2012 and 2019 in wild and captive koalas.

				KoRV subtype							
Origin	Region	#koalas	Total pol gene	A	B	D	F	G	H	I	Reference
QLD	northern (1891-1980s)	16		15 (94%)							Avila-Arcos *et al*. 2013
	Blair Athol	27	27 (100%)								Simmons *et al*. 2012
	SEQLD	250	250 (100%)								Simmons *et al*. 2012
	SEQLD	12		12 (100%)							Wedrowicz *et al*. 2018
	SEQLD	290		290 (100%)	83 (29%)						Quigley *et al*. 2018
	SEQLD	16		16 (100%)	4 (25%)	14 (88%)	4 (25%)	–	–	–	Quigley *et al*. 2019
	SEQLD	33		33 (100%)	33 (100%)	33 (100%)	–	11 (33%)	–	32 (97%)	Sarker *et al*. 2019
	SEQLD	18		18 (100%)	14 (78%)	17 (94%)	8 (44%)	2 (11%)	1 (6%)	1 (6%)	Chappell *et al*. 2017
NSW	NENSW	12		12 (100%)							Wedrowicz *et al*. 2018
	Pilliga	57	57 (100%)								Simmons *et al*. 2012
	Port Macquarie	15		15 (100%)							Wedrowicz *et al*. 2016
	Port Macquarie	43	43 (100%)								Simmons *et al*. 2012
VIC	Southern (1891-1980)	3		1 (33%)							Avila-Arcos *et al*. 2013
	Mallacoota	3		0 (0%)							Wedrowicz *et al*. 2018
	Raymond Island	136	38 (28%)								Legione *et al*. 2017
	Raymond Island	29	10 (35%)								Simmons *et al*. 2012
	Raymond Island	18		4 (22%)							Wedrowicz *et al*. 2018
	Greater Gippsland	33	6 (18%)								Legione *et al*. 2017
	Gippsland	20	11 (55%)								Simmons *et al*. 2012
	Strezlecki Ranges	26	18 (69%)								Simmons *et al*. 2012
	South Gippsland	203		64 (32%)							Wedrowicz *et al*. 2018
	South Gippsland/ Raymond Island	19		9 (47%)							Wedrowicz *et al*. 2016
	Central Gippsland	17		13 (76%)							Wedrowicz *et al*. 2018
	Snake Island	12	6 (50%)								Simmons *et al*. 2012
	French Island	94	23 (24%)								Legione *et al*. 2017
	French Island	28	6 (21%)								Simmons *et al*. 2012
	Phillip Island	6		0 (0%)							Wedrowicz *et al*. 2016
	Phillip Island	11	0 (0%)								Simmons *et al*. 2012
	Mornington Peninsula	15	4 (27%)								Legione *et al*. 2017
	South West Coast	178	30 (17%)								Legione *et al*. 2017
	Cape Otway	11		2 (18%)							Wedrowicz *et al*. 2018
	Far West	167	52 (31%)								Legione *et al*. 2017
	Far north Vic	15	6 (40%)								Legione *et al*. 2017
	General mainland	43	36 (82%)								Simmons *et al*. 2012
SA	Kangaroo Island	170		72 (42%)	0 (0%)						Fabijan *et al*. 2019a
	Kangaroo Island	162	24 (15%)								Simmons *et al*. 2012
	Mt Lofty	28		28 (100%)	28 (100%)	28 (100%)	–	12 (43%)	–	18 (64%)	Sarker *et al*. 2019
	Mt Lofty	75		49 (65%)	0 (0%)						Fabijan *et al*. 2019a
USA	Los Angeles Zoo	13		13(100%)	6(46%)						Xu *et al*. 2013
Japan	9 unnamed zoos: QLD/NSW koalas	40		40 (100%)	27 (68%)						Shojima *et al*. 2013b
	9 unnamed zoos: VIC koalas	11		4 (36%)	0 (0%)						Shojima *et al*. 2013b
	Kobe Oji Zoo, Saitama Children's Zoo, Hirakawa Zoological Park	20		20 (100%)	12 (60%)						Hashem *et al*. 2019; Kayesh *et al*. 2019
Germany	Duisburg Zoo	5		5 (100%)	2 (40%)						Fiebig, Keller and Denner 2016
Belgium	Antwerp Zoo	1		1 (100%)	1 (100%)						Fiebig, Keller and Denner 2016

Notes: Avila-Arcos*et al*.^2013^ data is from museum koala skins; Legione *et al*.^2017^total KoRV data was further tested to confirm 88% of cases were positive for KoRV-A while no KoRV-B was detected. Dashes indicate the subtype was not detected, blank spaces indicate the study did not test for the indicated target.

The biology of a typical *Gammaretrovirus* is well understood by virologists (Maclachlan and Dubovi 2010). Like all gammaretroviruses, KoRV is composed of a simple genome (∼8.5 kb long) with long terminal repeats (LTRs) at each end of a linear single-stranded RNA genome containing three genes; the group-specific antigen (*gag*) gene, the protease-polymerase (*pro-pol* or *pol*) gene and the envelope (*env*) gene (Fig. 6) (Maclachlan and Dubovi 2010; Denner and Young 2013). To understand if KoRV behaves the same way as other gammaretroviruses during infection, studies have investigated specific properties of KoRV genes and proteins.

The Gag protein, known to be important in virus budding from infected cells, was the first KoRV protein to be investigated. Using a constructed infectious clone of KoRV, researchers were able to show that, like other gammaretroviruses, Gag plays a critical role in KoRV budding by recruiting the endosomal sorting complexes required for transport (ESCRT) machinery through interaction with a specific L-domain region in KoRV Gag to allow virions to be released from infected cells (Shimode *et al*. 2013; Shojima *et al*.2013a). In addition, an alternative form of Gag, known as glycosylated Gag or glyco-gag, is known to be important for some gammaretroviruses to combat host restriction factors like APOBEC3 (Fig.   6). Interestingly, although the koala genome encodes for genes that appear to be APOBECs (XP_020850070.1, XP_020855279.1, XP_020849984.1 and XP_020819701.1), targeted investigation found no evidence that KoRV expresses glyco-gag (at least in human cell culture) and KoRV infectivity was restricted by both human APOBEC3G and mouse APOBEC3 (Nitta *et al*. 2015). In fact, the strong restriction of KoRV by human APOBEC3G, which is highly expressed in hematopoietic cells commonly targeted by gammaretroviruses, supports the assertion that there is a low likelihood of zoonotic transmission of KoRV to humans (Nitta *et al*. 2015). This assertion has so far held up, with no reports of veterinarians or koala animal carriers contracting a KoRV infection.

The other KoRV protein that has received targeted study has been the Env protein. Like other gammaretroviruses, KoRV is known to make two RNA transcripts from its genome; a full length genome transcript and an Env mRNA (Maclachlan and Dubovi 2010; Hobbs *et al*. 2014). Recent investigations have quantified the Env mRNA to be 5-fold more abundant than the unspliced genomic transcript (Yu *et al*. 2019). Within the Env protein, there are two regions; the surface (SU or gp70) protein followed by the transmembrane (TM or p15E) protein (Fig. 6). Within the TM protein, there are several epitopes known to be important for neutralizing antibody responses (the fusion peptide-proximal region (FPPR) and membrane-proximal external region (MPER)) (Fiebig *et al*. 2003), as well as a major immunosuppressive domain (IS or CKS-17 region) (Fig. 6) (Blinov *et al*. 2013). Studies of these important Env regions across the different KoRV subtypes and between northern and southern koalas have found striking conservation of these epitopes (Ishida *et al*.2015b; Olagoke *et al*. 2019). These data suggest that all KoRV subtypes may have similar immunosuppressive capability (at least based on CKS-17 interactions) and all subtypes may be targetable by the same neutralizing antibody responses. Together, this information provides a critical foundation for treatment options like vaccination (discussed below).

Another important area of KoRV biology that has advanced in recent years is our understanding of KoRV integration into the koala genome. Retroviruses in general must reverse transcribe and insert their genomes into the host genome (to become proviruses) in order to replicate and survive. Studies of koala genomes from the north have estimated KoRV proviral integration in the range of 133–165 copies per cell (Simmons *et al*. 2012; Hobbs *et al*. 2017; Johnson *et al*. 2018) while koala genomes from the south only appear to contain KoRV proviral integration at a rate of ∼1 copy/10 000 cells (Simmons *et al*. 2012). The vast majority of KoRV proviruses detected in individual koalas from the north appear to be endogenous in nature, with focused study and analysis of a northern koala family finding the proviral pattern detected in a joey could be accounted for by the proviral pattern of their parents (dam and sire) (Ishida *et al*.2015b). Additional studies, incorporating both modern wild koalas and museum koala samples dating back 130 years, have found that the sites of KoRV proviral integration were largely unique to each unrelated koala examined and were all KoRV-A in subtype (Tsangaras *et al*. 2014; Ishida *et al*. 2015a; Cui *et al*. 2016). This suggests that KoRV is in the early stages of retroviral invasion of the koala germline, with many unique insertion events proliferating at low frequency throughout the koala population (Ishida *et al*. 2015a). Finally, analysis of KoRV proviral LTR regions date initial KoRV endogenization as occurring no more than 22 200 – 49 900 years ago, although a much more recent time of integration is possible (Ishida *et al*. 2015a). This has created an exciting opportunity for virologists to study the process of viral endogenization in real time, as many other known mammalian ERVs integrated into their host's genome millions of years ago.

As with many viruses, variations of KoRV that appear to be defective in replication and/or transmission have also been detected (Xu *et al*. 2015; Hobbs *et al*. 2017; Sarker *et al*. 2020) (Quigley *et al*. 2020 under review). One study worth highlighting was a recent investigation of 97 southern Australian koalas that found KoRV proviral genes could be detected from 99% of koalas tested, but only 79% koalas had a complete detectable provirus (LTR, *gag*, *pol*, and *env*) and only 51% of koalas had detectable KoRV plasma RNA, suggesting that defective KoRV may be common in the south (Sarker *et al*. 2020). It should be noted that sequence missing from the *pol* gene of these defect proviruses is the same region targeted in common qPCR assay for KoRV (Tarlinton *et al*. 2005; Simmons *et al*. 2012) (Table 3B), raising questions about whether KoRV-negative results from southern koalas can really be taken as indicating KoRV-free koalas. Whether these defective KoRV variants have had any effect on the apparent lack of KoRV endogenization and prevalence in southern koalas remains an area of active investigation.

**Table 3B.** Tests used to determine KoRV infection prevalence.

Study reference	Gene target and KoRV subtype targeted (primers) for test	Reference for KoRV test
Avila-Arcos *et al*. 2013	gag-pol and env genes, KoRV-A (multiple sets)	Avila-Arcos *et al*. 2013
Simmons *et al*. 2012	pol gene, All KoRV (F/R) external set and internal set	Tarlinton *et al*. 2005; Simmons *et al*. 2012
Shojima *et al*.2013b	pol and env genes, KoRV-pol (F/R), KoRV-A (F/R), KoRV-J (F/R)	Shojima *et al*.2013b
Xu *et al*. 2013	env gene, KoRV-A (P3/P7), KoRV-B (P1/P5, P2/P4)	Xu *et al*. 2013
Fiebig, Keller and Denner 2016	env gene, KoRV-A (F/R), KoRV-B (P2/P4)	Fiebig, Keller and Denner 2016; Xu *et al*. 2013
Wedrowicz *et al*. 2016	env gene, KoRV-A (P3/P7)	Xu *et al*. 2013
Chappell *et al*. 2017	env gene, All KoRV (env22.F/env514.R)	Chappell *et al*. 2017
Legione *et al*. 2017	pol and env genes, All KoRV (F/R), KoRV-A (P3/P7), KoRV-B (P1/P5)	Tarlinton *et al*. 2005; Xu *et al*. 2013
Quigley *et al*. 2018	env gene, KoRV-A (UF/A_R), KoRV-B (UF/B_R)	Waugh *et al*. 2017
Wedrowicz *et al*. 2018	env gene, KoRV-A (P3/P7)	Xu *et al*. 2013
Fabijan *et al*. 2019a	pol gene, All KoRV (F/R) external set and internal set	Tarlinton *et al*. 2005; Simmons *et al*. 2012
Hashem *et al*. 2019; Kayesh *et al*. 2019	env gene, KoRV-A (UF/A_R), KoRV-B (UF/B_R)	Waugh *et al*. 2017
Quigley *et al*. 2019	env gene, All KoRV (env22.F/env514.R)	Chappell *et al*. 2017
Sarker *et al*. 2019	env gene, All KoRV (env22.F/env514.R)	Chappell *et al*. 2017

The last aspect of KoRV genetics that has been recently investigated has been its interaction with other ERVs in the koala genome. Studies to date have identified four ERVs in the koala genome, three of which appear to fall into established ERV families (ERV, ERVL and ERVK) and have been designated ERV.1, ERVL.1, ERVK.14 (Yu *et al*. 2019), and a fourth ERV designated Phascolarctos endogenous retroelement (PhER) (Hobbs *et al*. 2017). Detailed analysis has uncovered that KoRV and PhER have recombined at least 16 times to generate novel ERVs in the representative genome-sequenced koala, with the most prevalent version designated recKoRV1 (Hobbs *et al*. 2017; Lober *et al*. 2018). RecKoRV1 has been detected in koalas from across Australia, with a higher prevalence in northern koalas (Lober *et al*. 2018). Of special note, a unique case has been reported from two south Australian koalas, where recKoRV1 was detected in animals that had no detectable intact KoRV provirus (Lober *et al*. 2018). How this finding will fit with our current understanding of KoRV biology and detection is still an open question.

### Prevalence of KoRV in koalas

As KoRV gained recognition as an important koala pathogen to investigate, surveys of koala populations from across Australia have included KoRV detection (Fig. 8, Table 3). Between 2012 and 2019, KoRV prevalence has been determined in 10 studies of Australian koalas and four studies of koalas in international zoos (Table 3A) (Simmons *et al*. 2012; Avila-Arcos *et al*. 2013; Shojima *et al*.2013b; Fiebig, Keller and Denner 2016; Wedrowicz *et al*. 2016; Chappell *et al*. 2017; Legione *et al*. 2017; Quigley *et al*.2018a; Wedrowicz *et al*. 2018; Fabijan *et al*. 2019a; Hashem *et al*. 2019; Kayesh *et al*. 2019; Quigley *et al*. 2019; Sarker *et al*. 2019). As with *C. pecorum* detection, KoRV detection has relied on a range of PCR assays (Table 3B).

**Figure 9.**
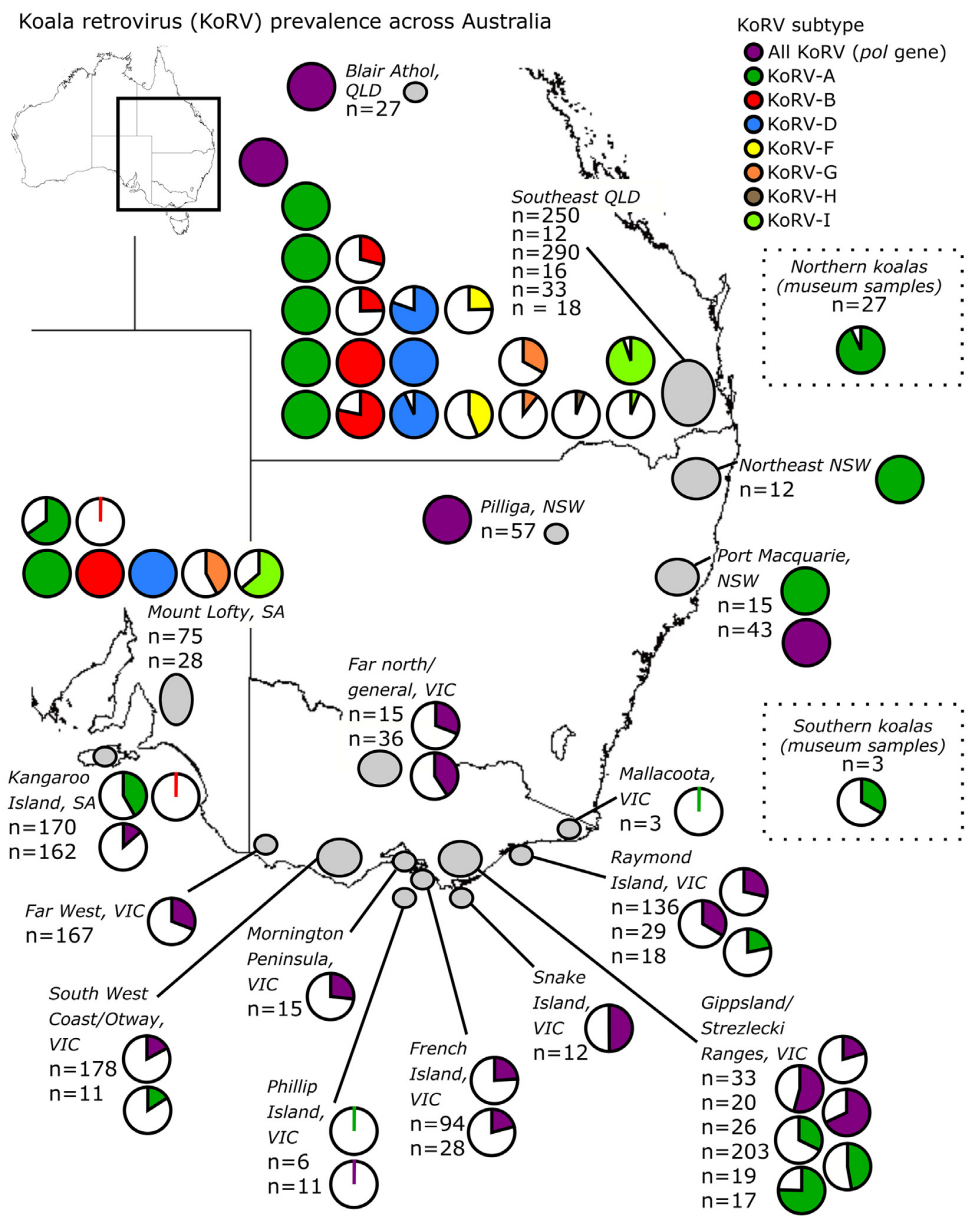
Prevalence of koala retrovirus (KoRV) provirus infection reported in studies from 2012 to 2019. Grey ellipticals indicate the mapped area investigated, pie charts represent the KoRV positivity by subtype reported in ‘n’ number of koalas tested. Details for each study can be found in Table 3.

Early studies, before the range of KoRV subtypes were recognized, focused on detecting KoRV using a general *pol* gene assay (now known to detect all the subtypes together) (Fig. 7). More recent studies have either combined KoRV *pol* gene pre-screening with specific KoRV-A and KoRV-B assays, have assayed directly for KoRV-A (and occasionally KoRV-B) or have surveyed for all KoRV subtypes simultaneously by deep amplicon sequencing (Figs 7 and 9, Table 3). These studies have found that northern koalas are always found to be infected with KoRV/KoRV-A (100% detectable infection) while southern koala infection rates range from 0% to 100%, depending on the area (Fig. 9, Table 3).

In northern koalas, beyond the endogenous 100% KoRV-A rate, believed exogenous subtype prevalence has been examined in southeast QLD. KoRV-B and KoRV-D appear to be the predominant non-KoRV-A subtypes, with KoRV-B detected in 25%-100% of koalas and KoRV-D detected in 88%-100% of koalas surveyed (Fig. 9, Table 3) (Chappell *et al*. 2017; Quigley *et al*. 2018a; Quigley *et al*. 2019; Sarker *et al*. 2019). Additional subtypes reported were not detected in all studies and included KoRV-F (25%-44% prevalence), KoRV-G (11%–33% prevalence), KoRV-H (6% prevalence) and KoRV-I (6%–97% prevalence) (Fig. 9, Table 3).

In the south, most studies have focused on total KoRV or KoRV-A detection alone. At one extreme, when more than five koalas were tested, Phillip Island was found to have a total KoRV or KoRV-A prevalence of 0% in two separate studies (Simmons *et al*. 2012; Wedrowicz *et al*. 2016). Although koala sampling numbers were not large (n = 6 and 11), this is the only island population of koalas where KoRV has not been detected. At the other extreme, a study of Mount Lofty, SA koalas used deep amplicon sequencing (a much more sensitive method) and found KoRV prevalence comparable to northern koalas, with all the koalas (n = 28) being KoRV-A, KoRV-B and KoRV-D positive (100%), KoRV-G with 43% prevalence and KoRV-I with 64% prevalence (Sarker *et al*. 2019). Through the rest of VIC and SA, the total KoRV or KoRV-A prevalence ranged from 17%–82% (Fig. 9, Table 3).

Finally, there have also been reports of KoRV prevalence from koalas in captive zoo populations internationally. Koalas in zoos appear to reflect the KoRV prevalence of their source region. Most international zoo koalas, including Los Angeles Zoo, USA, several Japanese zoos, Duisburg Zoo, Germany and Antwerp Zoo, Belgium, have KoRV prevalence patterns consistent with northern koalas (their koalas most likely origin), with 100% KoRV-A and 40%-68% KoRV-B (where there were at least five koalas) (Table 3) (Shojima *et al*. 2013b; Xu *et al*. 2013; Fiebig, Keller and Denner 2016; Hashem *et al*. 2019; Kayesh *et al*. 2019). Similarly, a collection of Japanese zoo koalas known to be from VIC had reported KoRV-A prevalence of only 36% and no KoRV-B detected, similar to current VIC wild koala numbers (Table 3) (Shojima *et al*. 2013b).

### Disease associated with KoRV and host response

Retroviruses, including *Gammaretrovirus* members, are the causative agents of certain types of cancers and immunosuppressive or immune-mediated diseases (Maclachlan and Dubovi 2010). In koalas, KoRV was originally identified because of its apparent link to leukemia (Canfield, Sabine and Love 1988) and this association has been maintained over time (Tarlinton *et al*. 2005; Ito *et al*. 2019). Additional links to immunosuppression, seen in associations between chlamydial disease rates and KoRV rates in koalas, have also been reported (Legione *et al*. 2017; Waugh *et al*. 2017; Quigley *et al*. 2018a). While reports continue to emerge showing KoRV in association with cancers and immunosuppressive outcomes in koalas, the data to date are of a correlative nature, not yet showing clear causation. However, as studies progress, clues into possible disease mechanisms of KoRV in koalas continue to be suggested.

With the division of KoRV into subtypes, KoRV-B has emerged as the major subtype often associated with lymphomas, leukemias and neoplasms in koalas (Xu *et al*. 2013; Quigley *et al*. 2018a). Although it is not clear why only KoRV-B has been associated with cancer in koalas, two biological features may offer hypotheses worthy of future investigation. The first feature is the point of distinction between KoRV subtypes found in the LTR region. The U3 region within the upstream LTR in KoRV contains an enhancer region for viral transcription (Fig. 7). Within KoRV-A, a single copy of this enhancer region is typically found, while the original KoRV-B strain was found to contain four copies of this enhancer region and a KoRV-F strain (now recognized as KoRV-D) has been found with five copies of this enhancer region (Xu *et al*. 2013, 2015). This suggested that non-KoRV-A strains may have higher transcription rates, a finding that was confirmed by comparing the proviral and expression loads of multiple KoRV subtypes in koalas over time (Quigley *et al*. 2019). Because KoRV randomly inserts into the host genome to replicate, increased viral transcription may lead to increased non-specific host transcription downstream of insertion and increased potential for oncogenic outcomes. However, this hypothesis would suggest that all non-KoRV-A subtypes, not just KoRV-B, should display increased oncogenic potential. A second biological feature that may separate KoRV-B and KoRV-D clade subtypes may be in the frequency of their defective variants. Defective KoRV-B and KoRV-D strains have been characterized (Xu *et al*. 2015), with the single koala genome available found to contain intact KoRV-B provirus but only detective KoRV-D and KoRV-E proviruses (Hobbs *et al*. 2017). Transmission studies have found that KoRV-B was consistently transmitted from dam-to-joey in both captive and wild settings (Xu *et al*. 2013; Quigley *et al*. 2018a) while the original KoRV-E and KoRV-F strains (now known to both represent KoRV-D/F strains) were not transmitted uniformly from either the dam- or sire-to-joey in a captive setting (Xu *et al*. 2015). The difference between these transmission rates is currently unknown, but if it is found that defective provirus is more common in the KoRV-D subtype clade, that may limit these subtypes in their ability to transcribe infectious particles and also limit the oncogenic potential of their U3 enhancer regions compared to KoRV-B. However, despite these interesting observations and hypotheses, the definitive reason for the increased oncogenic association of only KoRV-B is still an open research question.

Immunosuppression is the suppression of the immune system and its ability to fight infections. In koalas, the most prevalent infection by which to gauge immunosuppression is chlamydial disease. In the north, where KoRV-A is endogenous, koalas with detectable KoRV-B provirus showed a significant association with having chlamydial disease (Waugh *et al*. 2017; Quigley *et al*. 2018a). Cytokine investigation in northern koalas found a significant upregulation of IL-17A in KoRV-B-infected koalas (Maher and Higgins 2016), which corresponds with IL-17A as an immune marker for chlamydial disease severity and pathogenesis in northern koalas (Mathew *et al*. 2014). In the south, where KoRV-A appears to be exogenous, koalas with detectable KoRV-A provirus showed a significant association with having chlamydial disease (Legione *et al*. 2017). Interestingly, cytokine investigation in southern koalas found a significant downregulation of IL-17A and IFN-ɣ in KoRV positive koalas (by total KoRV *pol* gene assay) (Maher *et al*. 2019). Whether the discrepancy between IL-17A gene expression levels between northern and southern koalas can be explained by KoRV endogenization status, KoRV subtype infection profiles or some other non-KoRV related factor is an area of future investigation. Finally, chlamydial disease is not the only infection that KoRV appears to affect, with southern koalas also more likely to present with periodontitis (inflammation of the gums caused by bacteria in the dental plaque) when KoRV positive (by total KoRV *pol* gene assay) (Butcher *et al*. 2020).

Finally, the effect of KoRV expression over time on koala health has been investigated. While it had been previously shown that increases in total KoRV RNA levels in plasma were associated with the development of neoplasia (Tarlinton *et al*. 2005), details of KoRV subtype expression have only recently been investigated. Following a group of female northern koalas for multiple samplings over several years, koalas that remained healthy for the study (n = 11) were found to have stable KoRV-D/KoRV-A expression ratios while koalas that developed chlamydial cystitis (n = 5) had variable KoRV expression profiles, dominated by KoRV-A or KoRV-B and lacking KoRV-D, prior to disease onset (Quigley *et al*. 2019). Collectively, research has shown that different subtypes of KoRV have different effects and that both provirus and expressed virus contribute to pathology in koalas.

### Transmission of KoRV

Transmission of ERVs in the germline of the host genome, from parents to offspring, is a hallmark feature of endogenous retroviruses (Greenwood *et al*. 2018). As such, KoRV-A transmission in northern koalas follows the understood endogenous process. Transmission of the proposed exogenous variants (KoRV-B to -I in the north and KoRV-A to -I in the south) is an area of more uncertainty. As discussed in the disease section above, KoRV-B has been shown in both wild and captive koalas to be universally transmitted from dam-to-joey, with no apparent influence of the infection status of the sire (Xu *et al*. 2013; Quigley *et al*. 2018a), while KoRV-D strains do not show a predictable dam-to-joey or sire-to-joey transmission pattern (Xu *et al*. 2015). Dam-to-joey transmission is likely a significant transmission route for exogenous KoRV, as transcriptomic analysis of the koala mammary gland found KoRV transcripts to be 3% of all RNA in these cells (the fourth most abundant transcript detected) and proteomic analysis of koala milk found KoRV proteins (Gag, Pol and Env) to be ∼1% of all peptides in milk (collectively the fourteenth most abundant peptides) (Morris *et al*. 2016). Beyond mother to offspring transmission, the KoRV-B adult-to-adult contact transmission rate in a northern koala population with a general prevalence of 25% KoRV-B positivity was determined to be 3% new adult KoRV-B infections per year when 49 koalas were followed for three years by conventional proviral PCR (Quigley *et al*. 2018a).

Related to transmission has been the hypothesis that KoRV is currently spreading across Australia in a north to south direction. This hypothesis is based on the endogenous status of KoRV-A in the north (Tarlinton, Meers and Young 2006), similarity to other retroviruses that suggests KoRV may have entered the koala population from a spill-over event based in northern Australia (Greenwood *et al*. 2018) and prevalence surveys that show that KoRV has a 100% prevalence in the north, but not in the south (Tarlinton, Meers and Young 2006; Simmons *et al*. 2012). However, KoRV is clearly in southern Australia (Fig. 9), raising questions about why KoRV detection in the south is not higher. Whether the recent discoveries regarding the potential prevalence of defective KoRV strains, especially in southern koalas, can address this question, remains to be seen. However, with much still to learn about the receptors used by the KoRV-D subtype clade, there is still a lot to investigate regarding the transmission of KoRV in general and of different subtypes specifically.

### Detection of KoRV

Before 2012, when KoRV was considered a single entity, detection of a conserved region of the *pol* gene by PCR assay was the established method of total KoRV detection (Fig. 7) (Tarlinton *et al*. 2005). Since the definition of KoRV subtypes, several KoRV subtype-specific PCR assays targeting the *env* gene have been developed. Specific subtype primer sets have utilized a universal forward *env* gene primer with subtype-specific reverse primers based on the diversity in the VRA region of the *env* gene. KoRV-A and KoRV-B specific primer sets were originally designed based on published isolates (Xu *et al*. 2013; Waugh *et al*. 2017) and have recently been updated to capture more of the known diversity within these subtypes (Fig. 7) (Quigley *et al*. 2019). New assays to specifically detect KoRV-D and KoRV-F have also been designed and published (Fig. 7) (Quigley *et al*. 2019). Another very useful KoRV assay uses an *env* gene primer set (env22.F and env514.R) that spans either side of the VRA and allows for deep amplicon sequencing (similar to microbiome analysis for bacteria) of all KoRV subtypes present within a sample (Fig. 7) (Chappell *et al*. 2017). Finally, primer sets have also been designed to cover the complete KoRV genome (P8 and P9), allowing for long-range PCR of complete genomes or detection and analysis of defective variants (Fig. 7) (Xu *et al*. 2013, 2015).

### Treatment for KoRV – vaccines

Currently, there are no treatments available for KoRV in koalas. Antiretroviral drug therapies currently available for human retrovirus treatment require daily drug administration, a situation that is limiting for captive koala colonies and unfeasible in wild koala populations. As such, the field has turned to vaccine development as the best hope for a KoRV management strategy.

Before an animal is born, its immune system learns to recognize all the antigens that make up “self” and develops a tolerance to these to avoid auto-immunity. Given the endogenous nature of KoRV-A in northern koalas (meaning that KoRV-A antigens may be considered “self” as part of the genome), the first issue to be addressed was determining whether koalas produced, or could produce antibodies to KoRV. Initially, western blot analysis failed to detect KoRV antibodies from 16 captive and wild koalas (Fiebig *et al*.2015a). However, more detailed analysis using ELISA-based techniques from 235 wild koalas determined that the majority of koalas do naturally produce anti-KoRV antibodies, with anti-KoRV IgG levels peaking at approximately seven years of age and gradually declining thereafter (Olagoke *et al*. 2019). Additionally, some of these naturally occurring anti-KoRV antibodies mapped to known *Gammaretrovirus* neutralizing (and conserved) regions on the Env protein (FPPR, CSK-17 and MPER epitopes; Fig. 7), making it unsurprising that there was an inverse relationship between anti-KoRV IgG levels and circulating KoRV viral load (Olagoke *et al*. 2019). However, this raised the conundrum of how koalas produced antibodies to endogenous KoRV proteins that could be considered self-proteins. A possible explanation came with the discovery of KoRV antisense Piwi-interacting RNAs (piRNAs) in koalas. piRNAs guide the adaptive genome immune system to silence established transposons (and active ERVs) during germline development (Russell and LaMarre 2018; Ozata *et al*. 2019). Study within koalas has detected KoRV-A piRNAs apparently derived from isolated proviral insertions in the genome (Yu *et al*. 2019). This system, which is conserved from flies to mice, has the potential of silencing KoRV expression during fetal development, protecting developing joeys from KoRV and allowing KoRV proteins to be recognized as foreign in mature animals. These exciting findings set the stage for KoRV vaccine development.

The first KoRV vaccine trials tested whether recombinant KoRV (rKoRV) Env protein given to rats and goats could generate neutralizing antibodies (Denner 2013; Fiebig *et al*.2015b). These studies determined that binding antibodies could be generated to the FPPR and MPER regions of the Env protein, however, only the antibodies to the MPER region were neutralizing (Denner 2013; Fiebig *et al*. 2015b). This lead to a small safety trial where three northern koalas were given a rKoRV-A Env vaccine (Waugh *et al*.2016b). All three vaccinated koalas showed no signs of adverse reaction and the one koala that did not have detectable Env antibodies prior to vaccination was found to be generating Env antibodies post-vaccination (Waugh *et al*. 2016b). Subsequently, vaccination of six southern koalas also found no adverse vaccination effects, significant anti-KoRV antibody responses (including neutralizing antibody production) and decreases in KoRV circulating load post-vaccination (Olagoke *et al*. 2018). These promising pilot studies have established that vaccination for KoRV is safe in koalas and represents a promising avenue for KoRV management. The successes so far have paved the way for larger vaccine trials that are currently underway.

## FUTURE DIRECTIONS

With so many advances made to *C. pecorum* and KoRV understanding in the past eight years, the future of disease management in koalas has never looked more promising. The major opportunities for continued advancement continue along the same lines as the most significant recent advances – genetic understanding and improved treatments. Genome sequencing technologies, both at the metagenomic level for *C. pecorum* sequencing directly from clinical samples and additional koala genome sequencing for advanced KoRV study, are continuing to gain greater bioinformatics support and reduced costs. Advances in koala genome sequencing will also greatly improve our understanding of koala genetics, the genetic differences between northern and southern populations (where bottleneck effects are believed to have hindered the genetic robustness of southern koala populations) and how these host genetics affect *Chlamydia* and KoRV in the different regions. With similar methodologies, more detailed transcriptomic studies of both pathogens, in unique disease situations and over time, will further illuminate key markers of disease and key targets for therapeutic interventions. Greater pathogen understanding will be needed to develop new and improved treatment options. Alternatives to antibiotics for *C. pecorum*, such as a novel HtrA serine protease inhibitor (Lawrence *et al*. 2016), are in early development. Finally, effective vaccines for both *C. pecorum* and KoRV are in active development, with enormous potential to shift the disease landscape in the koala population. Australians, as well as animal-lovers from around the world, care about koalas and want to see these unique animals thrive. Continued progress in understanding and controlling the major diseases koalas suffer from is an actionable, obtainable goal that will hopefully allow many future generations to enjoy these marsupials in the wild.
